# Effects of repeated influenza vaccination and infection on durable seroprotection in healthcare workers

**DOI:** 10.1038/s41541-025-01259-x

**Published:** 2025-09-29

**Authors:** Mai-Chi Trieu, Amit Bansal, Marianne Sævik, Sonja Ljostveit, Åsne Jul-Larsen, Rebecca Jane Cox

**Affiliations:** 1 Influenza Centre, Department of Clinical Science, University of Bergen, Bergen, Norway; 2 Department of Microbiology, Haukeland University Hospital, Bergen, Norway; 3 Department of Medicine, Haukeland University Hospital, Bergen, Norway

**Keywords:** Antibodies, Influenza virus, Inactivated vaccines, Infection

## Abstract

Rapid evolution of seasonal influenza viruses necessitates annual vaccine reformulation to match circulating strains. Healthcare workers (HCWs) and high-risk groups are prioritised for annual influenza vaccination. However, repeated annual vaccination may affect immune protection. This study investigated the hemaglutination-inhibition (HI) antibody responses following influenza infection or repeated seasonal vaccination over four seasons in 250 HCWs with well-defined vaccination histories. Unvaccinated HCWs had high infection rates, with pre-existing antibodies providing protection. Infection or hybrid immunity generated higher antibody responses to A/H3N2 viruses than vaccination alone, whereas vaccination induced more durable A/H1N1 and B virus-specific antibodies. Vaccination boosted seroprotective antibodies, irrespective of previous vaccination histories. Moreover, repeated vaccination with the same virus for more than three consecutive seasons blunted antibody responses, while updating vaccine strains improved immunity. Annual influenza vaccination of HCWs should be strengthened to increase uptake, but next-generation influenza vaccines must improve vaccine immunogenicity, particularly against A/H3N2 viruses.

## Introduction

Influenza vaccination remains the most effective strategy to protect against influenza illness. Current seasonal vaccines are trivalent or quadrivalent, containing two influenza A strains (A/H1N1 and A/H3N2) and one or two influenza B strains (Victoria and/or Yamagata lineages). The rapid evolution of seasonal influenza viruses necessitates annual vaccine updates to match circulating viruses, ensuring immune protection. Annual influenza vaccination is recommended for healthcare workers (HCWs) who are at high risk of influenza infection^1^. Yet, the vaccination coverage rates in HCWs remain low across Europe (average 22%, range 5–58% in 2023/24)^2^, posing a risk for nosocomial infection. Influenza vaccine effectiveness (VE) is far from optimal, varying between seasons, age groups, and influenza types/subtypes^3^. Several factors contribute to the difficulties of optimising influenza vaccines such as annual updating of vaccine strains, antigenic similarity between vaccine antigens and circulating viruses, egg-adaption of vaccine viruses, and pre-existing immunity (review in ref. ^4^). Studies have raised concerns that repeated annual influenza vaccination may lead to reduced protection, especially for A/H3N2 viruses^5–7^. However, other studies have found no significant impact of previous vaccination on VE or antibody responses^5,8–11^. To date, the effect of repeated annual vaccination and/or infection on protection and antibody responses remains unclear.

Most licensed influenza vaccines induce antibodies against the major viral surface glycoprotein, hemagglutinin (HA) and to a lesser extent to neuraminidase (NA)^4^. The hemagglutination-inhibition (HI) assay is commonly used to quantify HA-specific antibodies that can prevent virus attachment to the host cell receptors. An HI titre ≥40 is associated with 50% protection against lab-confirmed influenza infection in adults^12,13^ and generally considered a correlate of protection. Whether this HI protective threshold applies to high-risk populations remains to be evaluated, as protection may vary by influenza viruses, age and across different populations^14,15^.

During the 2009 pandemic caused by a novel influenza A/H1N1 virus (A/H1N1pdm09), the use of adjuvants in pandemic vaccines allowed dose sparing and effectively prevented lab-confirmed influenza infections and hospitalisations^16^. In Norway, HCWs were prioritised to receive the AS03-adjuvanted pandemic vaccine in October 2009, preceding the peak of local pandemic activity^17^. From 2010/11 to 2016/17, the trivalent seasonal influenza vaccines (TIVs) contained the same A/H1N1pdm09 virus, while A/H3N2 and B components varied. This allowed us to study antibody responses after repeated vaccinations with the same^18^ or frequently updated viruses. In this study, we analysed influenza infection rates among unvaccinated HCWs over four seasons and evaluated the association between HI titres and seroprotection in HCWs. We further investigated antibody responses after natural infection and repeated vaccinations using HCWs’ historical and current seasonal vaccination status over four influenza seasons. Our study offers valuable insights into the longevity of antibody responses and the effects of repeated annual vaccinations, with important implications for influenza vaccination strategies.

## Results

**Fig. 1:**
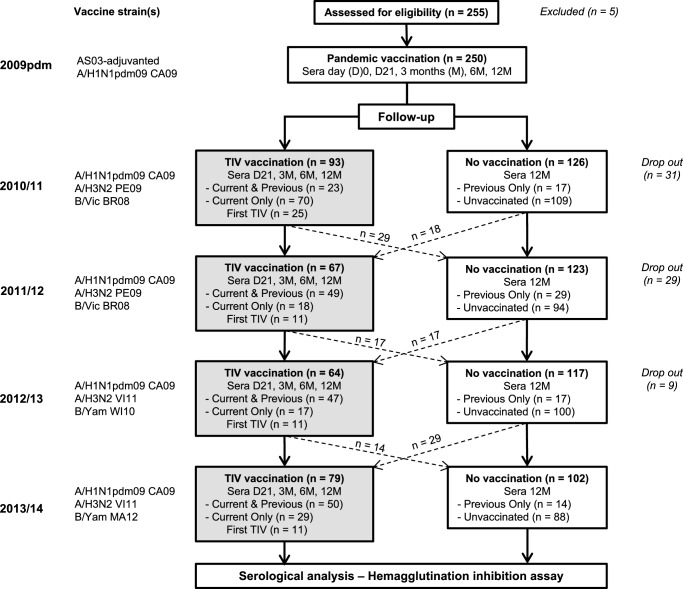
The study flow-chart. Healthcare workers (HCWs, n = 250) were vaccinated with the AS03-adjuvanted monovalent pandemic vaccine containing the A/California/7/2009(H1N1pdm09) (CA09) virus in an open-label clinical trial in 2009 and followed up for 5 years (European Clinical Trials Database, EudraCT 2009-016456-43; www.clinicaltrials.gov, NCT01003288)^17,18^. During four seasons from 2010/11 to 2013/14, HCWs voluntarily chose to be vaccinated with the trivalent seasonal influenza vaccine (TIV), containing the same A/H1N1pdm09 CA09 virus during the whole study period, while A/H3N2 and B viruses changed between seasons with vaccine updates: A/Perth/16/2009(H3N2) (PE09) and B/Brisbane/60/2008 (BR08) of B/Victoria lineage (B/Vic) in 2010/11 and 2011/12; A/Victoria/361/2011(H3N2) (VI11) in 2012/13 and 2013/14; B/Wisconsin/1/2010 (WI10) in 2012/13 and B/Massachusetts/2/2012 (MA12) in 2013/14, both belonging to B/Yamagata lineage (B/Yam). Blood samples were collected at day 21 (D21), 3, 6, and 12 months (3 M, 6 M, 12 M) post-vaccination in TIV-vaccinated HCWs and yearly pre-season in unvaccinated individuals. The dropout shows the total number of HCWs who dropped out from the study in each season, irrespective of seasonal vaccination status. In each season, HCWs were divided into 4 groups: Current & Previous (if they were vaccinated with TIVs in both the previous and the current seasons), Current Only (if they were only vaccinated in the current season), Previous Only (if they were only vaccinated in the previous season), and Unvaccinated (if they were not vaccinated in both the previous and the current seasons). The seasonal vaccination status in 2009 was obtained via questionnaire from the HCWs. See Supplementary Tables 5–8 for details. A subgroup of the Current only group who had never been previously vaccinated with TIV was termed First TIV, as this was their first recorded seasonal vaccination.

We recruited 250 HCWs, which corresponded to 929 person-years of follow-up. A total of 2562 serum samples were collected, including pre- and post-seasonal vaccination samples in vaccinated HCWs and yearly pre-season samples in unvaccinated HCWs. All 250 HCWs (78% female, median age 38 years old) received the AS03-adjuvanted pandemic A/H1N1pdm09 vaccine (Fig. 1), of whom 18% (45/250) received the seasonal TIV in 2009. Prior to 2009, 60% (150/250) of HCWs had a history of seasonal vaccination (Supplementary Table 1). Throughout the four seasons 2010/11–2013/14, 181 HCWs completed the study. The TIV uptake rates among HCWs were moderate, ranging between 35.3% and 42.5%. Only 18.2% (33/181) of HCWs were annually vaccinated in all four seasons, while 35.4% (64/181) were not immunised with TIV in any of the four seasons.

### High influenza infection rates among unvaccinated HCWs

We included all HCWs who were unvaccinated in one or more of the four seasons 2010/11–2013/14 (*n* = 159). Influenza infection was defined by seroconversion (≥4-fold increase in HI titres) between yearly pre-season samples against the circulating viruses (Table 1). We found 48.4% (77/159) of unvaccinated HCWs infected with a seasonal influenza virus between 2010 and 2014 (19.5% for A/H1N1pdm09, 17.6% for A/H3N2, and 30.2% for B), indicating high infection rates. The highest infection rates were observed in season 2010/11 for A/H1N1pdm09 (15.5%) and B (25.0%) viruses and in season 2011/12 for A/H3N2 (8.4%) viruses. Neither A/H1N1pdm09 nor B infections were found in season 2011/12. Infection with both influenza A and B viruses in one season occurred in 6.9% (11/159) of HCWs. One and 8 HCWs were infected with A/H1N1pdm09 and B viruses, respectively, in two different seasons. Strikingly, out of 64 HCWs who were unvaccinated in all four seasons, 71.9% (46/64) were infected with a seasonal virus between 2010 and 2014 (26.6% for A/H1N1pdm09, 23.4% for A/H3N2, and 48.4% for B). The infection patterns observed in our study aligned with the national influenza surveillance data (Supplementary Fig. 1). No consistent risk factor for influenza infection was found across the four seasons, except for the pre-season HI antibody titres <40 against the infecting viruses (Supplementary Tables 2–4).

**Table 1:** Influenza infection rates by seroconversion in unvaccinated healthcare workers

Type/subtype	2010/11	2011/12	2012/13	2013/14	Any season
Unvaccinated in any season^a^					
A/H1N1pdm09	18/116 (15.5%)	0/95 (0.0%)	8/99 (8.1%)	6/89 (6.7%)	31/159 (19.5%)^c^
A/H3N2	6/116 (5.2%)	8/95 (8.4%)	8/99 (8.1%)	6/89 (6.7%)	28/159 (17.6%)
B	29/116 (25.0%)	0/95 (0.0%)	13/99 (13.1%)	16/89 (18.0%)	48/159 (30.2%)^c^
Both A	3/116 (2.59%)	0/95 (0.0%)	2/99 (2.0%)	1/89 (1.1%)	6/159 (3.8%)
Both A and B	8/116 (6.9%)	0/95 (0.0%)	1/99 (1.0%)	2/89 (2.2%)	11/159 (6.9%)
Any influenza	42/116 (36.2%)	8/95 (8.4%)	26/99 (26.3%)	25/89 (28.1%)	77/159 (48.4%)^c^
Unvaccinated in all 4 seasons^b^					
A/H1N1pdm09	7/60 (11.7%)	0/54 (0.0%)	7/56 (12.5%)	4/59 (6.8%)	17/64 (26.6%)^c^
A/H3N2	2/60 (3.3%)	6/54 (11.1%)	3/56 (5.4%)	4/59 (6.8%)	15/64 (23.4%)
B	18/60 (30.0%)	0/54 (0.0%)	10/56 (17.9%)	11/59 (18.6%)	31/64 (48.4%)^c^
Both A	0/60 (0.0%)	0/54 (0.0%)	1/56 (1.8%)	1/59 (1.7%)	2/64 (3.1%)
Both A and B	3/60 (5.0%)	0/54 (0.0%)	1/56 (1.8%)	1/59 (1.7%)	5/64 (7.8%)
Any influenza	24/60 (40.0%)	6/54 (11.1%)	18/56 (32.1%)	17/59 (28.8%)	46/64 (71.9%)^c^

Data are presented as number/ total number (%).

^a^In each season, HCWs who chose not to receive the trivalent seasonal influenza vaccines (TIVs) were considered unvaccinated regardless of their vaccination status in prior season and were included in the unvaccinated HCWs in any season. Some HCWs changed their vaccination status between seasons (see Fig. 1 for detail).

^b^The same cohort of HCWs who were only vaccinated with the pandemic A/H1N1pdm09 vaccine in 2009 and received no TIVs throughout 4 seasons 2010/11–2013/14.

^c^HCWs who were reinfected with the same type/subtype or infection with any influenza virus in >1 seasons were counted once.

### Pre-existing HI antibodies correlated with protection

**Fig. 2:**
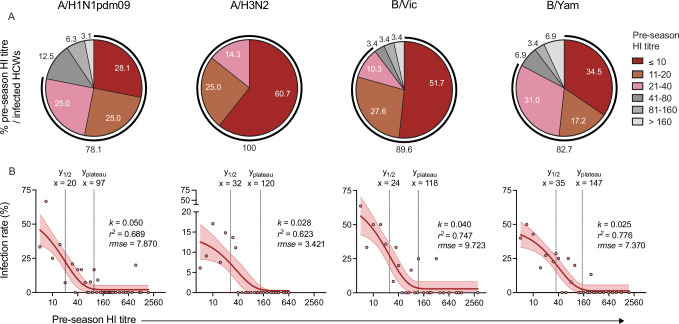
Pre-existing hemagglutination-inhibition (HI) titres were correlated with protection in unvaccinated healthcare workers. Infection was defined among 159 unvaccinated HCWs by seroconversion (≥ 4-fold increase in HI titres) between two consecutive yearly samples (A/H1N1pdm09 *n* = 32, A/H3N2 *n* = 28, B/Victoria (B/Vic) *n* = 29, or B/Yamagata (B/Yam) *n* = 29). See Table 1 for details. **A** The percentage (%) of infected HCWs stratified by the pre-season HI titres as pie chart. Exact percentages are shown. The outer lines indicate the total percentages of HCWs with pre-season HI titres ≤ 40 among infected HCWs. **B** Correlation between infection rates and pre-season HI titres against A/H1N1pdm09, A/H3N2, B/Vic and B/Yam viruses. Symbols show infection rates (% infected/total unvaccinated HCWs) that were calculated for each pre-season HI titre using combined 4-season HI data from paired pre- and post-season serum samples (A/H1N1pdm09 *n* = 304, A/H3N2 *n* = 399, B/Vic *n* = 116, or B/Yam *n* = 188). The y-axis scale for A/H3N2 differs from that of A/H1N1pdm09 and B viruses. Data were fitted with an exponential plateau non-linear regression line with 95% confidence intervals. The estimated values of *x* at *y*_*1/2*_ (50% reduction from the starting y), *x* at *y*_*plateau*_, the rate constant *k*, the coefficient of variance *r*^2^, and the root mean square error (*rmse*) are reported.

We further investigated the association between HI titres and protection against influenza A or B infection in HCWs. Our analysis included 159 HCWs who were unvaccinated in any season and excluded vaccinated HCWs due to the inability to induce seroconversion among these vaccinees. Pre-season paired HI titres (A/H1N1pdm09 *n* = 304, A/H3N2 *n* = 399, B/Victoria *n* = 116, or B/Yamagata *n* = 188) and all infection incidences throughout four seasons 2010/11–2013/14 were utilised. HI data from season 2011/12 were excluded from analysis of A/H1N1pdm09 and B/ Victoria viruses, as no infection with these viruses was detected. Among infected HCWs, the majority had pre-season HI titres ≤40 against the infecting viruses: 78.1% (25/32) for A/H1N1pdm09, 100% (28/28) for A/H3N2, 89.7% (26/29) for B/Victoria and 82.8% (24/29) for B/Yamagata viruses (Fig. 2A). We calculated the infection rates corresponding to each pre-season HI titre and data were best fitted with an exponential-plateau non-linear regression model for each virus (Fig. 2B). A progressive decrease in infection rates was observed with increasing pre-season HI titres against each infecting virus, followed by a plateau of 0.5–2% infection rates when the pre-season HI titre reached 97 for A/H1N1pdm09, 120 for A/H3N2, 118 for B/Victoria, and 147 for B/Yamagata viruses. HI titres of 20 against A/H1N1pdm09, 32 against A/H3N2, 24 against B/Victoria, and 35 against B/Yamagata viruses corresponded to 50% reduction in infection rates. Comparable results were found in a sensitivity analysis including pre-season HI data and infection rates of 64 HCWs who remained unvaccinated in all four seasons 2010/11–2013/14 (Supplementary Fig. 2). In this four-season unvaccinated cohort, the infection rates plateaued with the pre-season HI titres of 77 for A/H1N1pdm09, 83 for A/H3N2, 137 for B/Victoria, and 127 for B/Yamagata viruses. HI titres of 18 against A/H1N1pdm09, 24 against A/H3N2, 30 against B/Victoria, and 23 against B/Yamagata viruses were associated with 50% reduction in infection rates. Overall, an HI titre of 40 corresponded to 79%, 59%, 72%, and 56% protection in unvaccinated HCWs against A/H1N1pdm09, A/H3N2, B/Victoria, and B/Yamagata infection, respectively.

The strict infection criteria ≥4-fold increase in HI titres between yearly samples likely underestimated the actual burden of influenza, particularly in view of antibody waning throughout the year and high pre-existing titres. Therefore, we explored two alternative infection criteria to examine the association between HI titres and protection: (1) > 2-fold increase for high pre-season titres ≥80 and ≥4-fold increase for pre-season titres <80, (2) >2-fold increase for all pre-season titres. We found additional 5 HCWs with high pre-season titres ≥80 and >2-fold rise against A/H1N1 virus in season 2010, and 1 HCW against B/Yam in season 2013. A total of 8 HCWs with pre-season titres <80 and >2-fold rise against A/H1N1, 2 against A/H3N2, 5 against B/Vic and 8 against B/Yam across four seasons were observed. The infection rates were recalculated, and data were then fitted with exponential-plateau non-linear regression models for each virus. Surprisingly, similar results were found between infection criteria using either >2-fold increase for high pre-season titres only (Supplementary Fig. 3) or all pre-season titres (Supplementary Fig. 4) or ≥4-fold increase (Fig. 2). Thus, our data demonstrated pre-existing HI titres as correlate of protection in HCWs.

### Higher A/H3N2-specific antibodies after infection compared to vaccination

**Fig. 3:**
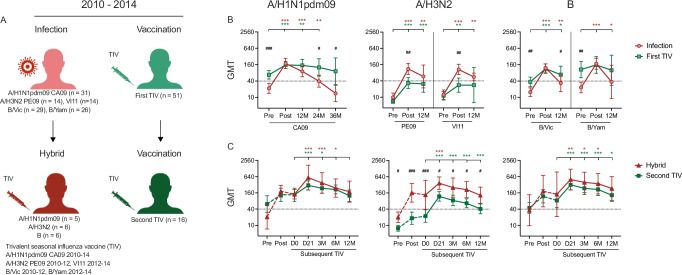
The hemagglutination-inhibition (HI) antibody responses after influenza infection and vaccination. During 2010/11–2013/14, the circulating viruses in Norway matched the vaccine viruses included in the trivalent seasonal influenza vaccine (TIV): A/California/7/2009(H1N1pdm09) (CA09), A/Perth/16/2009(H3N2) (PE09) or A/Victoria/361/2011(H3N2) (VI11), and B viruses belonging to B/Victoria (B/Vic) or B/Yamagata (B/Yam) lineage. **A** Study population. Healthcare workers (HCWs) were either infected (A/H1N1pdm09 *n* = 31, A/H3N2 PE09 *n* = 14 or VI11 *n* = 14, B/Vic *n* = 29, B/Yam *n* = 26) or vaccinated with TIV for the first time (First TIV, *n* = 51) in any of the 4 seasons. See Table 1 and Fig. 1 for details. A subgroup of infected HCWs (Hybrid; A/H1N1pdm09 *n* = 5, any A/H3N2 *n* = 6, and any B *n* = 6) and first-TIV vaccinated HCWs (Second TIV, *n* = 16) chose to be vaccinated in the subsequent season. **B** Comparisons of the magnitude and the durability of HI antibodies after infection or the first TIV. The geometric mean HI titres (GMTs) with 95% confidence interval (CI) are shown for pre- and post-season, and 12–36 months (M) later. The dotted line indicates the protective threshold HI titre of 40. **C** Comparisons of HI antibody responses to subsequent TIV in HCWs with hybrid immunity or vaccination immunity alone. The GMTs with 95% CI are shown for pre- and post-season of infection or first vaccination, and pre- day (D)0 and post-subsequent TIV at 21 days, 3–12 months (M). The effect of exposure (Infection vs. First TIV, Hybrid vs. Second TIV) on log-transformed HI titres was analysed in mixed-effect models, followed by post-hoc tests comparing estimated means with Holm-Sidak’s multiple comparison adjustment (Supplementary Tables 9 and 10). Within each group (*), the estimated means at pre-season time points were compared to post-season, 12-, 24-, and 36-month time points (B) and the estimated means at day 0 were compared to post-subsequent TIV time points (C). The estimated means at each time point were also compared between groups (^#^) (Infection vs. First TIV (B), Hybrid vs. Second TIV (C)). Levels of significance are shown above the graph with colours referring to groups. *^,#^*p* < 0.05. **^,##^*p* < 0.01. ^***,###^*p* < 0.001.

We hypothesised that influenza infection induces higher and more durable antibody responses compared to vaccination. To examine this, we included HI titres after the first detected infection from all infected HCWs (A/H1N1pdm09 *n* = 31, A/H3N2 *n* = 28, B/Victoria *n* = 29, B/Yamagata *n* = 26 excluding 3 s infections) and HI titres after the first recorded vaccination from all first-time vaccinees (First TIV, *n* = 51), irrespective of the season of infection or vaccination (Fig. 3A). We found that A/H3N2 infection generated significantly higher HI titres than vaccination (GMT post-PE09 infection 111 vs. vaccination 33, *p* = 0.001; post-VI11 infection 106 vs. vaccination 28, *p* = 0.002) (Fig. 3B). The A/H3N2-specific GMTs remained above the protective HI titre of 40 up to 12 months after infection, whereas GMTs were <40 by 12 months post-vaccination. Contradicting our hypothesis, infection induced similar but less durable HI antibodies against A/H1N1pdm09 and B viruses compared to vaccination. The post-vaccination GMTs were significantly higher than post-infection at 24 (*p* = 0.026) and 36 (*p* = 0.024) months against A/H1N1pdm09 virus and 12 months against B/Victoria virus (*p* = 0.048).

A small group of infected HCWs and first-time vaccinees chose to be vaccinated with TIVs in the following season and were referred to as Hybrid (A/H1N1pdm09-infected *n* = 5, A/H3N2-infected *n* = 6, any B-infected *n* = 6) and Second TIV (*n* = 16) subgroups, respectively (Fig. 3A). The subsequent TIV significantly boosted HI titres in both the Hybrid and the Second TIV subgroups despite their relatively high pre-vaccination titres (Fig. 3C). No difference in the GMTs against A/H1N1pdm09 and B viruses was found between the two subgroups at any time point. In contrast, the GMTs against A/H3N2 viruses were higher in the Hybrid than the Second TIV subgroup at all time points (*p* < 0.05). This emphasises that hybrid immunity from infection and vaccination was more effective at inducing A/H3N2-specific antibodies than vaccination alone.

### TIVs boosted seroprotective HI antibodies irrespective of prior vaccination

**Fig. 4:**
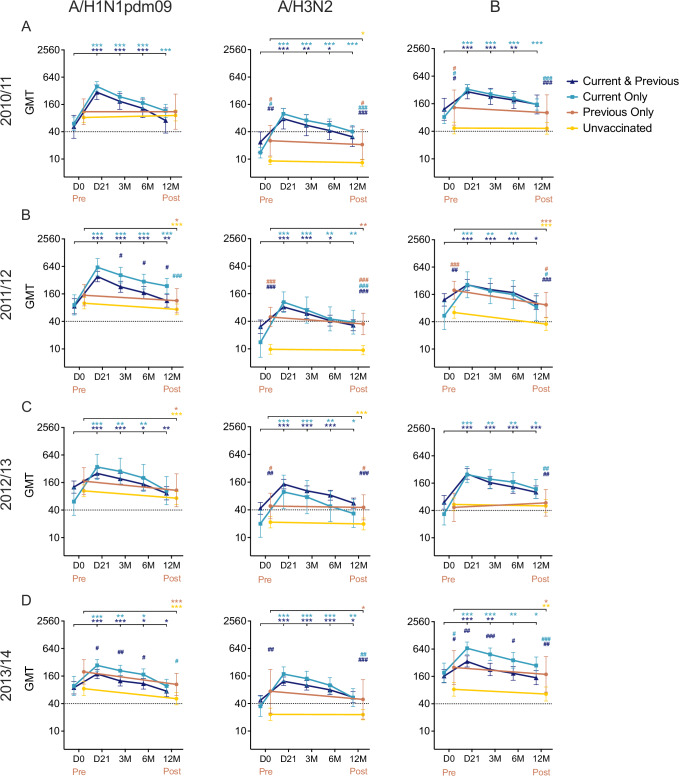
The hemagglutination-inhibition (HI) antibody responses after seasonal vaccination. Healthcare workers (HCWs) were divided into 4 groups: vaccinated with the trivalent seasonal influenza vaccines (TIVs) in both the current and previous seasons (Current and Previous), vaccinated either in the current season (Current Only) or the previous season only (Previous Only), and unvaccinated in both seasons (Unvaccinated). See Fig. 1 for details. The HI antibody responses against A/H1N1pdm09, A/H3N2, and B viruses in 2010/11 (**A**), 2011/12 (**B**), 2012/13 (**C**), and 2013/14 (**D**). The TIVs contained the same A/California/7/2009(H1N1pdm09) virus during 2010–14, while A/H3N2 and B viruses changed from A/Perth/16/2009(H3N2) and B/Brisbane/60/2008 Victoria lineage during 2010–12 to A/Victoria/361/2011(H3N2) and B/Wisconsin/1/2010 or B/Massachusetts/2/2012 Yamagata lineage during 2012–14. The geometric mean HI titres (GMT) are shown with 95% confidence interval (CI) pre-vaccination day (D)0 and post-vaccination at 21 days, 3, 6 and 12 months (M) or pre- and post-season. The dotted line indicates the protective threshold HI titre of 40. The effect of vaccination status on log-transformed HI titres was examined in mixed-effect models, followed by post-hoc tests comparing estimated means with Holm-Sidak’s multiple comparison adjustment (Supplementary Table 11). Within each group (*), the estimated means at pre-vaccination/season time points were compared to post-vaccination/season time points. The estimated means at each time point were also compared between the Unvaccinated group and the other three groups (^#^). Levels of significance are shown above the graph with colours referring to groups. *^,#^*p* < 0.05. **^,##^*p* < 0.01. ***^,###^*p* < 0.001.

We further evaluated the HI antibody responses to TIVs by comparing four groups of HCWs with differing vaccination status in each season: vaccinated with TIVs in both the current and previous seasons (Current and Previous), vaccinated in the current season only (Current Only) or the previous season only (Previous Only), or unvaccinated in both seasons (Unvaccinated) (Fig. 1). Infected HCWs were excluded from the time of infection onwards. Demographic analysis revealed no significant difference in age and sex distribution among the groups in each season (Supplementary Tables 5–8). Upon vaccination, HI antibodies were increased to similar levels between the Current and Previous and the Current Only groups (Fig. 4). Exceptions were observed against A/H1N1pdm09 virus in 2011/12 and 2013/14 and B virus in 2013/14, where the Current Only group had significantly higher GMTs post-vaccination than the Current and Previous group (Fig. 4B, D). HI titres against A/H1N1pdm09 virus were comparable among the four groups and remained >40 throughout one season, likely due to all HCWs having been primed with the adjuvanted pandemic A/H1N1pdm09 vaccine in 2009. The Previous Only group had comparable GMTs against A/H3N2 and B viruses to the two Current and Previous and Current Only groups. The A/H3N2 and B virus-specific GMTs were lower in the Unvaccinated group than the three vaccinated groups, although only significant for the Current and Previous group in all four seasons (*p* < 0.05). This indicates that TIVs effectively boosted high levels of HI antibodies against all three vaccine viruses and maintained A/H3N2- and B-specific antibody levels beyond a single season.

**Fig. 5:**
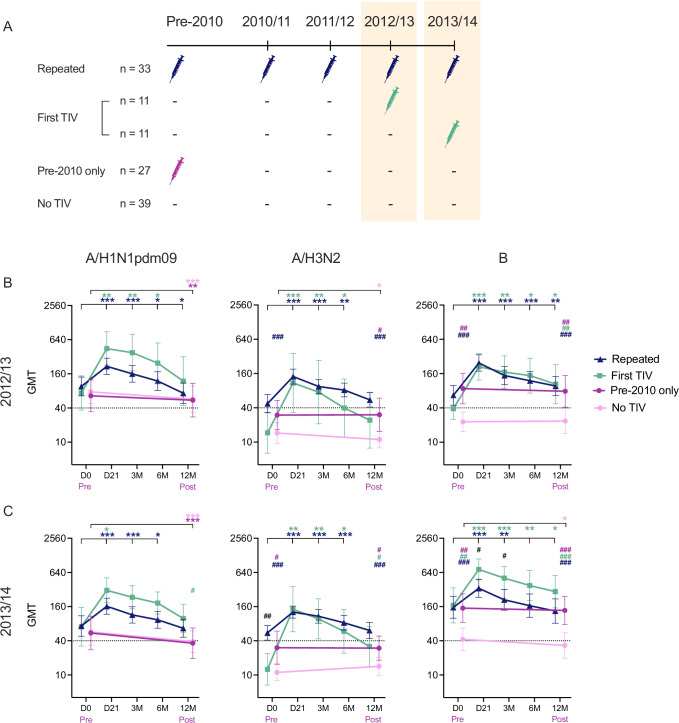
Higher hemagglutination-inhibition (HI) titres in healthcare workers vaccinated with seasonal vaccines than those unvaccinated. **A** Cohorts to study the long-term effect of repeated vaccination. Healthcare workers (HCWs) were retrospectively divided into 4 cohort: repeatedly vaccinated with the trivalent seasonal influenza vaccines (TIVs) in all 4 seasons 2010–14 and before 2010 (Repeated, *n* = 33), vaccinated for the first time (First TIV) in 2012/13 (*n* = 11) or 2013/14 (*n* = 11), vaccinated before 2010 only (Pre-2010 Only, *n* = 27), and unvaccinated in all 4 seasons without prior TIV vaccination (No TIV, *n* = 39). The HI titres against A/H1N1pdm09, A/H3N2, and B viruses in the 4 cohorts in season 2012/13 (**B**) and 2013/14 (**C**). The TIV contained the same A/California/7/2009(H1N1pdm09) and A/Victoria/361/2011(H3N2) viruses during 2012–14, while B viruses changed from B/Wisconsin/1/2010 in 2012/13 to B/Massachusetts/2/2012 in 2013/14, both belonging to the B/Yamagata lineage. The geometric mean HI titres (GMT) are shown with 95% confidence interval (CI) pre-vaccination day (D)0 and post-vaccination at 21 days, 3, 6 and 12 months (M) or pre- and post-season. The dotted line indicates the protective threshold HI titre of 40. The effect of vaccination status on log-transformed HI titres was examined in linear mixed-effect models, followed by post-hoc tests comparing estimated means with Holm-Sidak’s multiple comparison adjustment (Supplementary Table 12). Within each group (*), the estimated means at pre-vaccination/season time points were compared to post-vaccination/season time points. The estimated means at each time point were also compared between the No TIV group and the other three groups (^#^). Levels of significance are shown above the graph with colours referring to groups. *^,#^*p* < 0.05. **^,##^*p* < 0.01. ^***,###^*p* < 0.001.

To assess the effect of prior TIVs on antibody responses, we examined the HI titres in four HCW cohorts, who were strictly defined by vaccination histories across three-four consecutive seasons: repeatedly vaccinated with TIVs in all four seasons 2010/11–2013/14 and before 2010 (Repeated, *n* = 33), first vaccinated in either 2012/13 or 2013/14 (First TIV, *n* = 11 each season), vaccinated before 2010 only (Pre-2010 Only, *n* = 27), and unvaccinated in all four seasons and before 2010 (No TIV, *n* = 39) (Fig. 5A). Similarly, TIVs boosted HI antibodies to comparable levels between HCWs who received TIV for the first time or repeatedly (Fig. 5B, C). The First TIV cohort had significantly higher GMTs post-vaccination than the Repeated cohort only against B virus in 2013/14 (both 21 days, 3 months *p* = 0.048) (Fig. 5C). We observed similar GMTs against A/H1N1pdm09 virus in all four cohorts. The A/H1N1pdm09-specific HI antibodies only decreased to <40 in the Pre-2010 Only and the No TIV cohorts by the end of season 2013/14 (equivalent to 60 months post-2009 pandemic vaccination). The GMTs against A/H3N2 and B viruses in the Pre-2010 Only cohort were comparable to the Repeated and the First TIV cohorts at the end of season and significantly higher than the No TIV cohort both pre- and post-season. Overall, this suggests a beneficial effect of TIVs in boosting durable HI antibodies against A/H3N2 and B viruses and further extends the antibody durability after receipt of TIVs to beyond four seasons.

### Increased antibody persistence after repeated vaccination with the same viruses

**Fig. 6:**
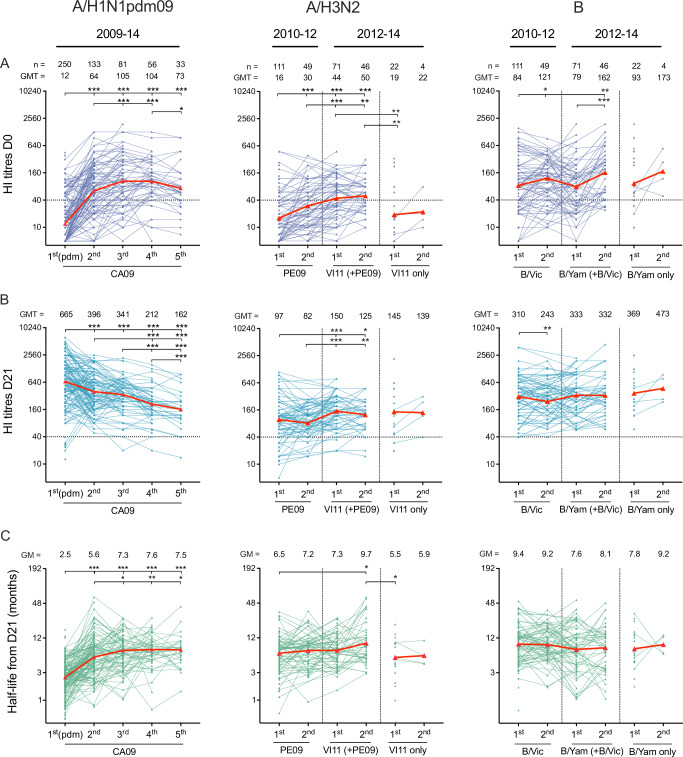
The 5-year dynamic of antibody responses after repeated vaccinations against the same influenza A viruses or B lineages in healthcare workers. All healthcare workers (HCWs) were vaccinated with the AS03-adjuvanted pandemic vaccine containing the A/California/7/2009(H1N1pdm09) (CA09) virus in 2009. During 2010–14, the trivalent seasonal influenza vaccines (TIVs) included the same A/H1N1pdm09 CA09 strain, while the A/H3N2 and B components changed from A/Perth/16/2009(H3N2) (PE09) and B/Brisbane/60/2008 of B/Victoria lineage (B/Vic) during 2010–12 to A/Victoria/361/2011(H3N2) (VI11) and B/Wisconsin/1/2010 or B/Massachusetts/2/2012 of B/Yamagata lineage (B/Yam) during 2012–14. The dynamics of the pre-vaccination HI titres (**A**), the peak HI titres at 21 days post-vaccination (**B**), and the estimated half-life of the peak titres post-vaccination by months (**C**) are presented by the sequential order of vaccination against the same influenza A viruses or B lineages. They are designated as 1st, 2nd, 3rd, 4th, and 5th vaccination for A/H1N1pdm09 CA09 virus; 1st and 2nd PE09, 1st and 2nd VI11 with previous vaccination against PE09 for A/H3N2 viruses; and 1st and 2nd B/Vic, 1st and 2nd B/Yam with previous vaccination against B/Vic for B viruses. HCWs who were vaccinated with TIV in 2012–14 were designated as 1st and 2nd VI11 only or 1st and 2nd B/Yam only. Symbols with connecting lines represent an individual’s response. The symbols with connecting lines in red indicate the geometric means (GM) of all HCWs. The dotted line represents the protective threshold HI titre of 40. The numbers of HCWs (*n*), the geometric mean titres (GMT), and the GM are reported above the graph. The effect of repeated vaccination on log-transformed HI titres was analysed in mixed-effect models, followed by post-hoc tests comparing estimated means between the sequential order of vaccinations with Holm-Sidak’s multiple comparison adjustment (Supplementary Table 13). Levels of significance are shown above the graph. *p < 0.05. **p < 0.01. ***p < 0.001.

We investigated the effect of repeated vaccinations with the same vaccine strains on HI antibody responses. For A/H1N1pdm09 virus, HI titres were evaluated after five repeated vaccinations with the same A/California/7/2009(H1N1pdm09) (CA09) strain, including the first adjuvanted pandemic vaccination in 2009 and subsequent non-adjuvanted seasonal TIVs. Following repeated A/H1N1pdm09 CA09 vaccination, the baseline pre-vaccination HI titres significantly increased (*p* < 0.001) and plateaued between the third and fifth vaccinations, whereas the peak HI titres 21 days post-vaccination significantly decreased (*p* < 0.001) to the lowest peak titres after the fifth vaccination (Fig. 6A, B). This resulted in the lowest antibody fold-rises observed after the fourth and fifth CA09 vaccinations (both geometric mean (GM) 2.1-fold) (Supplementary Fig. 5).

For A/H3N2 and B viruses, we could only examine HI titres after the first and second vaccinations since the vaccine components changed from A/Perth/16/2009(H3N2) (PE09) virus and B/Victoria lineage in 2010/11 and 2011/12 to A/Victoria/361/2011(H3N2) (VI11) virus and B/Yamagata lineage in 2012/13 and 2013/14. Similarly, a significant increase in baseline pre-vaccination HI titres following repeated A/H3N2 or B vaccination was found (*p* < 0.05) (Fig. 6A). HCWs who vaccinated with both A/H3N2 PE09 and VI11 viruses had significantly higher pre-vaccination HI titres than HCWs who vaccinated with VI11 virus only (*p* = 0.002), suggesting a collateral benefit of previous PE09 vaccination on HI titres against VI11 virus. No collateral benefit of previous B/Victoria vaccination on HI titres against B/Yamagata virus was observed. Updating vaccine strains led to significantly higher peak HI titres for A/H3N2 virus (GMT second PE09 82 vs. first VI11 (+PE09) 150; *p* < 0.001) (Fig. 6B) and higher antibody fold-rise post-vaccination for B virus (GM second B/Victoria 2.0-fold vs. first B/Yamagata (+B/Victoria) 4.4-fold; *p* = 0.007) (Supplementary Fig. 5).

To evaluate antibody durability following repeated vaccinations, individual HI data were log-transformed and fitted with a linear regression model, and the antibody half-life waning from the peak HI titres 21 days post-vaccination was estimated from the model. For A/H1N1pdm09 virus, antibody half-life gradually increased and plateaued between the third and fifth vaccinations (GM first CA09 vaccination 2.5 months vs. third-fifth vaccination 7.3–7.6 months; *p* < 0.001), suggesting an increase in antibody persistence after repeated vaccinations (Fig. 6C). In HCWs vaccinated with both A/H3N2 PE09 and VI11 viruses, antibody half-life significantly increased from the first PE09 vaccination to the second VI11 vaccination (GM 6.5 vs. 9.7 months; *p* = 0.011), indicating an increase in antibody durability after three or more A/H3N2 vaccinations. No evidence of increasing antibody half-life with repeated vaccinations was observed for B viruses. Overall, these data demonstrate that repeated vaccinations resulted in antibodies reaching homoeostasis levels and further vaccination with the same viruses did not increase antibodies.

## Discussion

In this longitudinal HCW study, we investigated the real-world impact of repeated, previous or current TIV on seroprotective antibody responses and infection rates with 929 person-years follow-up. During our four-season study, there was one strain update for A/H3N2 and two for the B strains, whilst the A/H1Npdm09 strain remained the same throughout the study. We found that antibody induction after vaccination was the highest when the vaccine strain was first introduced, although antibodies after the first A/H1N1pdm09 vaccination may be biased by the introduction of a novel pandemic strain and the adjuvant effect in the 2009 pandemic vaccine^17–19^. Furthermore, updating the vaccine strains increased antibody fold-rise after vaccination, although TIVs boosted antibodies even in previously vaccinated individuals. The A/H3N2 strain evolves rapidly and requires more frequent vaccine updates^4^. We found higher antibody responses after A/H3N2 infection or hybrid immunity than vaccination alone, emphasising the necessity for improved influenza vaccines. We further showed very high rates of infection in unvaccinated HCWs and calculated that HI titres of approximately 40 provide >50% protection from infection during a season. Overall, our study has implications for influenza vaccination policies in HCWs and vaccine strain selection strategies.

Our findings highlight the need to improve vaccine immunogenicity, particularly against A/H3N2 viruses, as in our HCW cohort HI titres against A/H3N2 viruses waned to <40 by 6–12 months post-TIV, whereas HI titres against A/H1N1 and B viruses remained ≥40 at 12 months post-TIV. Importantly, certain age groups are more susceptible to A/H3N2 viruses according to the imprinting theory that influenza virus exposures during childhood can affect antibody responses later in life^20–23^. A recent study reported that middle-aged individuals preferentially generated non-neutralising antibodies against recently circulating A/H3N2 viruses of the 3C2.A clade which emerged in 2014/15, thus was susceptible to A/H3N2 infection even with seasonal vaccination^22^. Vaccine production in embryonated eggs can introduce egg-adapted changes in A/H3N2 viruses^24–26^, which may in part explain the declining VE for A/H3N2 viruses in the past decade^27,28^. Other vaccine platforms (e.g. cell culture) or the use of adjuvants may be favourable in next-generation influenza vaccines to improve vaccine immunogenicity. Additionally, strategies for shortening the vaccine development process by utilising new platforms such as mRNA and delaying strain selection closer to the start of influenza season may be beneficial for improving VE against A/H3N2. Recent reports from phase 1/2 trials of an mRNA influenza vaccine showed promising results with higher and broader antibody responses than a licensed inactivated influenza vaccine^29,30^. Another strategy is to incorporate multiple A/H3N2 strains, including one antigenically advanced strain, in seasonal vaccines to produce both strain-specific and cross-reactive antibodies^31,32^, thus increasing vaccine immunogenicity and protection for A/H3N2 viruses. Based on our data, we recommend continuing and strengthening uptake of annual influenza vaccination in HCWs while better vaccine strategies are developed, including mitigation for the potential effect of influenza virus imprinting.

The effects of repeated annual influenza vaccination on antibody responses and protection are not fully understood. A systematic review found that people vaccinated in two consecutive seasons had lower VE than those vaccinated in the current season only, but higher VE than those vaccinated in the previous season only^7^. A recent longitudinal study reported that individuals vaccinated >2 consecutive seasons had lower post-vaccination HI titres, regardless of age, than individuals unvaccinated in the previous two seasons before the current vaccination^33^. Here, we found no consistent difference in antibody responses between repeated vaccinees and those with fewer vaccinations, suggesting no difference in seroprotection among HCWs with varying vaccination histories. In agreement, repeated vaccination was found to improve antibody responses to drifted strains emerging after the vaccine viruses are selected^34^. We demonstrated that being vaccinated with seasonal vaccines, even several years ago, improved antibody levels and seroprotection against A/H3N2 and B viruses. This suggests that TIVs are protective against infection by boosting higher antibody levels and maintaining them above the protective levels beyond a single season. Our findings that repeated annual vaccination with the same viruses increased baseline antibodies pre-vaccination agreed with other studies^35–39^. We further extend these observations that the highest baseline antibody levels are achieved after 2–3 vaccinations, resulting in the lowest antibody fold-changes post-vaccination after 4–5 vaccinations. We observed a gradual decrease in antibody levels post-vaccination after each sequential vaccination with the same A/H1N1pdm09 virus, which has not been described before^40,41^. This implies that repeated vaccination may unintendedly blunt antibody responses in specific instances, such as repeated vaccination against the same virus for more than three consecutive seasons. In agreement, one study showed a decline in plasmablast responses after the second and up to the fourth annual vaccination^42^. Simultaneously, vaccine-induced antibodies waned at a slower rate with successive vaccinations as reported in a previous study^40^. The highest antibody half-life post-TIV was after three vaccinations. This indicates that while antibodies steadily rise with repeated exposures, they eventually reach a homoeostatic level, leading to a blunted response to further vaccinations. One explanation could be due to pre-existing immunity. High pre-vaccination antibodies were correlated with reduced plasmablast activation, perhaps by blocking vaccine epitopes, which may limit B- and T-cell stimulation or memory B cell activation^35,43–45^. Further investigation is needed on the mechanisms behind the long-term maintenance and stabilisation of antibody responses and the effect of repeated vaccination with the same or different viruses on protection.

Our study’s strength lies in the comprehensive vaccination history of participants and longitudinal follow-up with frequent sampling over four seasons. This enabled a robust dynamic assessment of antibody responses following influenza infection and repeated vaccination. We took advantage of annual blood sampling before influenza season and determined infection by seroconversion. We report high influenza infection rates among unvaccinated HCWs, likely underestimating the actual burden due to stringent infection criteria (≥4-fold increase in HI titres between yearly samples despite high pre-existing titres or antibody waning throughout the year). Unlike most studies of influenza infection that only have post-infection samples, we had pre-infection samples and were able to evaluate HI titres associated with protection. HI titres ≥32 or ≥40 are considered correlates of protection, associated with 50% protection against lab-confirmed influenza infection in adults^12,13^. However, this correlation varies by influenza viruses, age and populations^14,15^. Here, we found HI titres ≥20 against A/H1N1pdm09, ≥32 against A/H3N2, ≥24 against B/Victoria, and ≥35 against B/Yamagata viruses were associated with 50% protection against seroconversion in HCWs. This would include protection against both clinical and subclinical infections, not only lab-confirmed infections. HCWs are at three times higher risk of influenza infection than the general population^1^. Most HCWs would have influenza-specific memory T and B cells and cross-reactive antibodies from previous infections that potentially reduce the HI titres needed for protection. Thus, both serology and molecular tests and other immunological correlates, including T- and B-cell responses, are needed for evaluating vaccine efficacy and effectiveness and for broader evaluation of vaccine protection against clinical and subclinical infection.

Inherent in longitudinal cohort studies, we faced challenges such as participants lost to long-term follow-up, changes in decision to be vaccinated and missing samples. However, HCWs are a valuable group with good records of influenza vaccination histories and self-reported influenza-like illness. Our data from 2010 to 2014 remains important as we explored the complexity of influenza exposure in adults and antibody responses following infection and vaccination over four consecutive seasons. The study is highly relevant for future vaccine strategies, as the influenza epidemic situation has not changed, and as we return to trivalent vaccines^46^. A limitation is the lack of data on lab-confirmed infection, as testing was not routine in the hospital and was only used for severely ill patients. Studies demonstrate boosting of HA-specific antibodies against previously circulating and future influenza viruses after influenza infection and vaccination^31–33,47^, which were not assessed in this study. Further investigation is warranted to determine the potential effect of influenza virus imprinting^20–23^ on antibody responses. The effect of repeated annual vaccination with the A/H1N1pdm09 was preceded by an adjuvanted pandemic vaccination; thus, different results may occur in populations immunised with a non-adjuvanted pandemic vaccine. Previous studies suggested differing effects of repeated vaccination due to antigenic differences between vaccine strains and circulating viruses^48^. Our findings should be interpreted with caution as the vaccine viruses matched with the circulating viruses during the study period^49^. Moreover, studies indicate blunted binding antibody responses^50^, increased HA head-directed antibodies vs. no change in stem-directed antibodies^51^, improved antibody quality^36,38,52^, reduced plasmablasts^42^, and maintained memory B and T cells^53–55^ after repeated vaccination, which were not measured in this study. Further studies are required to evaluate immune response following repeated vaccinations with the same or different strains in high-risk populations. We recommend a multi-season longitudinal study design, particularly random controlled trials^56^, along with routine inclusion of influenza vaccination history and broader immune analyses for better evaluation of the long-term effect of repeated annual vaccination.

In conclusion, seasonal influenza vaccination was effective in preventing influenza infection and boosting antibodies to above seroprotective levels in HCWs. Repeated annual vaccination increased antibody persistence, which correlated with lower antibody responses to subsequent vaccinations. Repeated vaccinations with the same viruses more than thrice blunted antibody responses to vaccines, whereas updating the vaccine viruses likely improved antibody responses to vaccines. Our findings highlight the need to improve vaccine immunogenicity, particularly against A/H3N2 viruses, and demonstrate that repeated annual vaccination may have complex effects on antibody responses. More research is required to conclusively determine whether repeated annual vaccination has any negative effect on protection and the mechanism behind. We support continuing the current recommendation of annual influenza vaccination in HCWs. However, more refined vaccine strategies are essential to mitigate any potential negative effects of repeated vaccination while maintaining the benefits of seasonal vaccines.

## Methods

### Study population and sampling

HCWs (*n* = 250) all received an AS03-adjuvanted pandemic vaccine in October–December 2009. This study population was based on a single-arm clinical trial of an AS03-adjuvanted pandemic vaccine in HCWs at Haukeland University Hospital (Norway) in 2009. The study was approved by the regional ethics committee (REKVest-2012/1772) and the Norwegian Medicines Agency (ClinicalTrials.gov NCT01003288)^17,18^. Although annual influenza vaccination was recommended and free of charge, seasonal vaccination was voluntary during 2010 and 2014 (Fig. 1). Participants provided written informed consent before inclusion and follow-up. Demographic information and seasonal vaccination status before and in 2009 were obtained through questionnaires.

Clotted blood samples were collected pre-vaccination (day 0), and at 21 days, 3, 6, and 12 months after pandemic vaccination or after each TIV. In HCWs who chose not to be vaccinated with TIVs, blood samples were collected annually prior to each influenza season. Sera were separated, aliquoted and stored at −80 °C until analysed. The first samples were collected in October 2009, and the last samples in October 2014.

### Vaccines

The 2009 AS03-adjuvanted monovalent pandemic inactivated vaccine contained 3.75 μg hemagglutinin (HA) of A/California/7/2009(H1N1pdm09) (CA09) virus (Pandemrix, GlaxoSmithKline, Belgium). From 2010/11 to 2013/14, the standard-dose non-adjuvanted TIVs, either subunit (Influvac, Abbott Laboratories) or split virion (Vaxigrip, Sanofi Pasteur), were used, containing 15 μg HA per strain. The A/H1N1 component was the pandemic CA09 virus during the whole study period, while the A/H3N2 and B components differed between seasons: A/Perth/16/2009(H3N2) (PE09) and B/Brisbane/60/2008 (BR08) of Victoria lineage in both 2010/11 and 2011/12; A/Victoria/361/2011(H3N2) (VI11) in both 2012/13 and 2013/14; B/Wisconsin/1/2010 (WI10) in 2012/13 and B/Massachusetts/2/2012 (MA12) in 2013/14, both of Yamagata lineage (Fig. 1).

### Hemagglutination inhibition (HI) assay

Sera were treated with receptor-destroying enzyme (Seiken, Japan) and pre-adsorbed with turkey red blood cells (TRBC) before testing in duplicates. Serial dilutions of sera were incubated with beta-propiolactone(BPL)-inactivated influenza A viruses or ether-extracted influenza B viruses (National Institute for Biological Standards and Control, UK; Influenza Reagent Resources, USA) and TRBC as previously described^17,18,57^. The HI titre was the reciprocal of the highest serum dilution that inhibited hemagglutination. Titres <10 were assigned a value of 5 for calculation purposes. Geometric mean HI titres (GMT) were calculated from replicates and used as final titres for each participant. Seroprotection referred to HI titres ≥40, and seroconversion referred to a ≥4-fold increase in HI titres between two time points.

### Statistical analyses

Infection rates (%) were calculated among unvaccinated HCWs as “the number of infected HCWs”/”total number of HCWs”*100 for each virus, double infection, and any infection in each season or across four seasons. To assess protection by pre-existing HI titres in unvaccinated HCWs, the infection rates (%) were stratified by pre-season HI titres for each virus and calculated as “the number of infected HCWs”/“total number of HCWs who had the same pre-season HI titres”*100. Data was best fitted with an exponential plateau non-linear regression model. The equation was *y* *=* *y*_*max*_*-* *(y*_*max*_*-* *y*_*0*_)*exp(-k* *x)*; where *y* is the dependent variable, *y*_*0*_ is the starting population, *y*_*max*_ is the maximum value that *y* can reach as *x* increases, and *k* is the rate constant inverse of *x* time units. Linear mixed-effect models accounting for the subject variance with repeated measures (random effect) were used to evaluate the effects of exposure (infection vs. vaccination, vaccination groups), time/season (fixed effects) and their interactions on log-transformed HI titres. Post-hoc analysis was performed to compare between time points within each group or between groups at each time point with Holm-Sidak’s multiple comparison tests. To calculate antibody half-life after vaccination, a linear regression model was fitted for each individual’s log-transformed HI data at 21 days, 3, 6 and 12 months post-vaccination. The intercepts and slopes from the regression models were used to estimate the half-life waning from the peak HI titres at 21 days post-vaccination. Demographic and clinical characteristics of the study population were examined by Pearson’s chi-squared tests, except for age, which was examined by Kruskal–Wallis test. *P* values < 0.05 indicated statistical significance. Analyses were performed in GraphPad Prism version 10.4.0 for macOS (GraphPad software) and R version 4.4.1.

## Supplementary information

Trieu MC Supplementary file

## Data Availability

Small participant subgroups increase the risk of identifying sensitive data for individual patients; consequently, the data are not publicly accessible. However, the datasets generated during the current study are available upon reasonable requests to the corresponding authors.

## Code availability

Our analyses utilised GraphPad Prism version 10.4.0 and R version 4.4.1. using R packages *gtsummary* (available from: https://www.danieldsjoberg.com/gtsummary/), stats (available from https://rdrr.io/r/stats/stats-package.html), nlme (https://cran.r-project.org/web/packages/nlme/index.html), and lme4 (available from https://cran.r-project.org/web/packages/lme4/index.html).
