# Supplementary material for: Effects of repeated influenza vaccination and infection on durable seroprotection in healthcare workers
Source: NPJ Vaccines. 2025 Sep 29;10:213. doi: 10.1038/s41541-025-01259-x (PMC12480258; doi:10.1038/s41541-025-01259-x)
Supplement: Supplementary file 1 — Trieu MC Supplementary file [file 41541_2025_1259_MOESM1_ESM.pdf]

Effects of repeated influenza vaccination and infection on durable seroprotection in healthcare workers

Mai-Chi Trieu[1.3.*]. Amit Bansal[1]. Marianne Sævik[2]. Sonja Ljostveit[1]. Åsne Jul-Larsen[1]. Rebecca Jane Cox[1.3.*]

[1]*Influenza Centre. Department of Clinical Science. University of Bergen. Bergen. Norway;* [2]*Department of Medicine. and* [3]*Department of Microbiology. Haukeland University Hospital. Bergen. Norway*

Supplementary file

Supplementary Figures

Supplementary Figure 1: Distribution of circulating influenza viruses in Norway during the study period 2009/10-2013/14. 1
Supplementary Figure 2: Pre-existing hemagglutination-inhibition (HI) titres correlated with protection in healthcare workers who were unvaccinated throughout 4 seasons 2010/11-2013/14. .. 2
Supplementary Figure 3: Pre-existing hemagglutination-inhibition (HI) titres correlated with protection in healthcare workers using alternative infection criteria for high titres. 3
Supplementary Figure 4: Pre-existing hemagglutination-inhibition (HI) titres correlated with protection in healthcare workers using alternative infection criteria for all titres. 4
Supplementary Figure 5: The five-year dynamic of hemagglutination-inhibition (HI) antibody fold-change after repeated vaccinations against the same influenza A viruses or B lineages in healthcare workers 5

Supplementary Tables

Supplementary Table 1: Demographics and clinical characteristics of the study cohort. 6
Supplementary Table 2: Demographics and clinical characteristics of influenza A/H1N1pdm09-infected and uninfected healthcare workers. 7
Supplementary Table 3: Demographics and clinical characteristics of influenza A/H3N2-infected and uninfected healthcare workers. 8
Supplementary Table 4: Demographics and clinical characteristics of influenza B-infected and uninfected healthcare workers. 9
Supplementary Table 5: Demographics and clinical characteristics of healthcare workers in 2010/11. 10
Supplementary Table 6: Demographics and clinical characteristics of healthcare workers in 2011/12. 11
Supplementary Table 7: Demographics and clinical characteristics of healthcare workers in 2012/13. 12
Supplementary Table 8: Demographics and clinical characteristics of healthcare workers in 2013/14. 13
Supplementary Table 9: Statistical analysis for Figure 3B. 14
Supplementary Table 10: Statistical analysis for Figure 3C. 16
Supplementary Table 11: Statistical analysis for Figure 4. 17
Supplementary Table 12: Statistical analysis for Figure 5. 22
Supplementary Table 13: Statistical analysis for Figure 6. 25

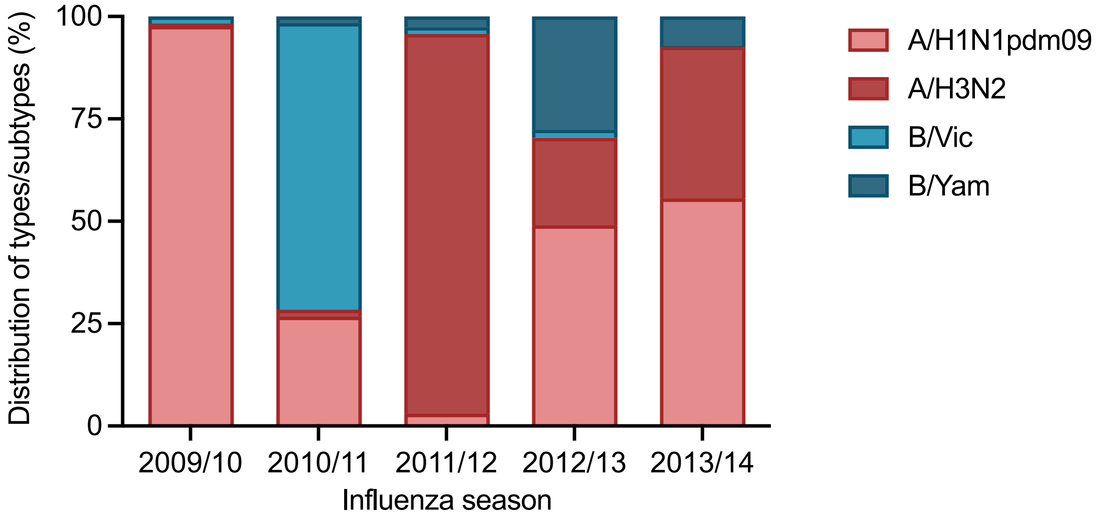

Supplementary Figure 1: Distribution of circulating influenza viruses in Norway during the study period 2009/10-2013/14.

The distribution of circulating influenza types/subtypes varies each season in Norway. The pandemic A/H1N1pdm09 virus emerged and dominated the season 2009/10 and replaced the previous seasonal A/H1N1 viruses to circulate in later seasons. Influenza A/H1N1pdm09 subtype and B/Victoria (B/Vic) lineage dominated the 2010/11 season. while A/H3N2 subtype dominated the 2011/12 season. In the latter 2 seasons 2012/13 and 2013/14. both influenza A/H1N1pdm09 and A/H3N2 subtypes co-circulated. while B/Yamagata (B/Yam) was the dominated B lineage. The infection rates in unvaccinated healthcare workers (HCWs) in our study (see Table 1 for details) corresponded to these surveillance data. Source: sentinel influenza surveillance data. provided by Dr. Olav Hungnes. WHO National Influenza Centre. Norwegian Institute of Public Health. Oslo. Norway.

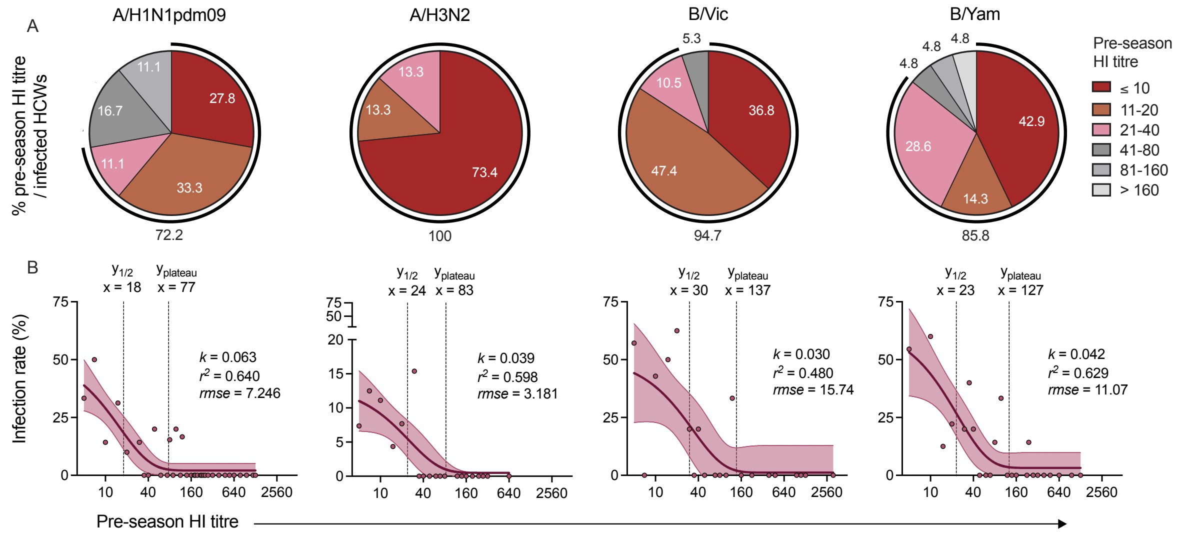

Supplementary Figure 2: Pre-existing hemagglutination-inhibition (HI) titres correlated with protection in healthcare workers who were unvaccinated throughout 4 seasons 2010/11-2013/14.

Healthcare workers (HCWs) who were only vaccinated with the pandemic A/H1N1pdm09 vaccine in 2009 and had no seasonal immunisations throughout 2010/11-2013/14 (n = 64) are included in this analysis. Infection was defined by seroconversion (≥ 4-fold increase in HI titres) between two consecutive yearly samples (A/H1N1pdm09 n = 18. A/H3N2 n = 15. B/Victoria (B/Vic) n = 19. or B/Yamagata (B/Yam) n = 21). See Table 1 for details. (A) The percentage (%) of infected HCWs stratified by the pre-season HI titres as pie chart. Exact percentages are shown. The outer lines indicate the total percentages of HCWs with pre-season HI titres ≤ 40 among infected HCWs. (B) Correlation between infection rates and pre-season HI titres against A/H1N1pdm09. A/H3N2. B/Vic. and B/Yam viruses. Symbols show infection rates (% infected/total HCWs) that were calculated for each pre-season HI titre using combined four-season HI data from paired pre- and post-season serum samples (A/H1N1pdm09 n = 175. A/H3N2 n = 229. B/Vic n = 60. or B/Yam n = 115). Data were fitted with an exponential plateau non-linear regression line with 95% confidence intervals. The estimated values of x at $y_{1/2}$ (50% reduction from the starting y). x at $y_{plateau}$. the rate constant k. the coefficient of variance r^2. and the root mean square error (*rmse*) are reported.

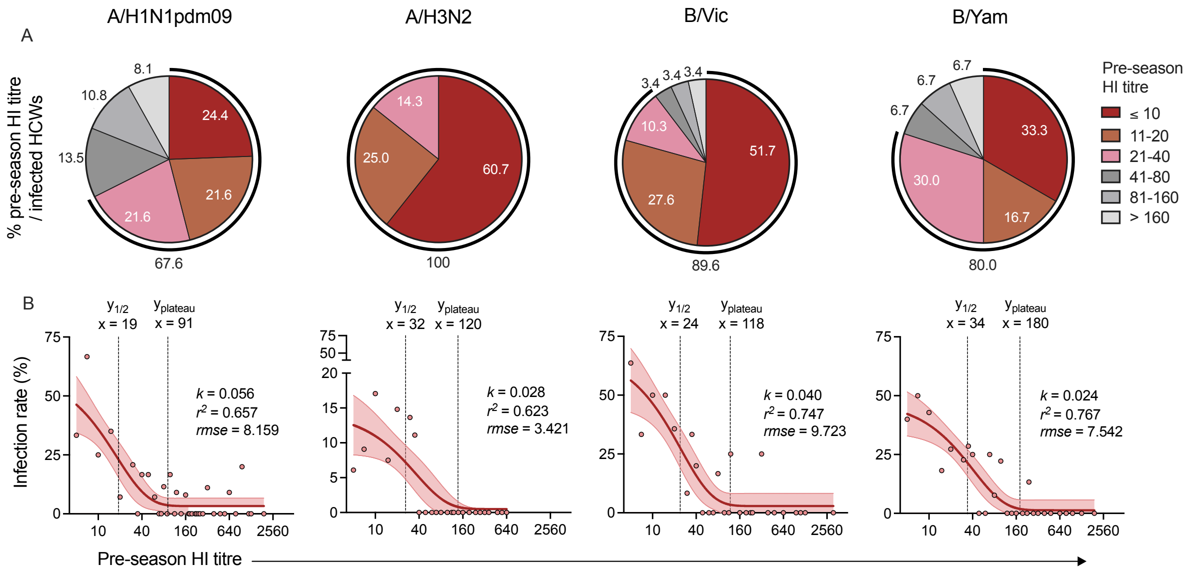

Supplementary Figure 3: Pre-existing hemagglutination-inhibition (HI) titres correlated with protection in healthcare workers using alternative infection criteria for high titres.

Infection was defined among 159 unvaccinated HCWs by alternative high-titre criteria: > 2-fold increase between two consecutive yearly samples for high pre-season HI titres ≥ 80 and ≥ 4-fold increase for pre-season HI titres HI titre < 80. We found additional 5 HCWs with high pre-season titres ≥ 80 and > 2-fold rise against A/H1N1 virus in season 2010. and 1 HCW against B/Yamagata (B/Yam) in season 2013. No additional infection using alternative high-titre infection criteria was found for A/H1N1 and B/Yam viruses in other seasons as well as A/H3N2 and B/Victoria (B/Vic) viruses in all 4 seasons. (A) The percentage (%) of infected HCWs stratified by the pre-season HI titres as pie chart (A/H1N1pdm09 n = 37. A/H3N2 n = 28. B/Vic n = 29. or B/Yam n = 30). Exact percentages are shown. The outer lines indicate the total percentages of HCWs with pre-season HI titres ≤ 40 among infected HCWs. (B) Correlation between infection rates and pre-season HI titres against A/H1N1pdm09. A/H3N2. B/Vic and B/Yam viruses. Symbols show infection rates (% infected/total unvaccinated HCWs) that were calculated for each pre-season HI titre using combined 4-season HI data from paired pre- and post-season serum samples (A/H1N1pdm09 n = 304. A/H3N2 n = 399. B/Vic n = 116. or B/Yam n = 188). Data were fitted with an exponential plateau non-linear regression line with 95% confidence intervals. The estimated values of *x* at $y_{1/2}$ (50% reduction from the starting *y*). *x* at $y_{plateau}$. the rate constant *k*. the coefficient of variance r^2. and the root mean square error (*rmse*) are reported.

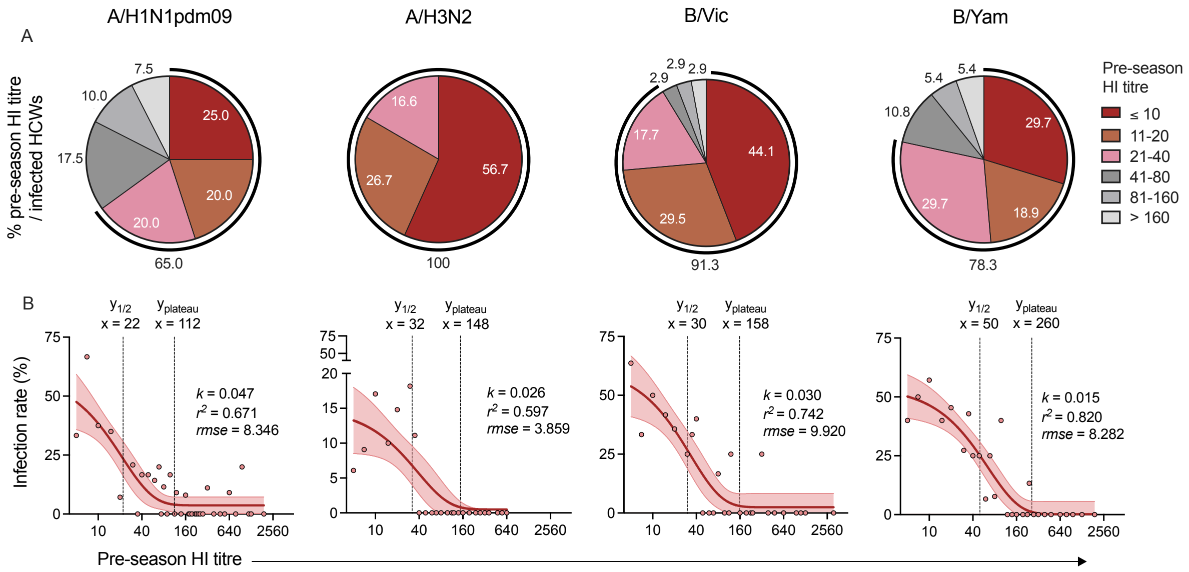

Supplementary Figure 4: Pre-existing hemagglutination-inhibition (HI) titres correlated with protection in healthcare workers using alternative infection criteria for all titres.

Infection was defined among 159 unvaccinated HCWs by alternative all-titre criteria: > 2-fold increase between two consecutive yearly samples for all pre-season HI titres. We found additional 13 HCWs with > 2-fold rise against A/H1N1. 2 HCWs against A/H3N2. 5 HCWs against B/Victoria (B/Vic) and 9 HCWs against B/Yamagata (B/Yam) across 4 seasons compared to the original infection criteria ≥ 4-fold increase. (A) The percentage (%) of infected HCWs stratified by the pre-season HI titres as pie chart (A/H1N1pdm09 n = 45. A/H3N2 n = 30. B/Vic n = 34. or B/Yam n = 38). Exact percentages are shown. The outer lines indicate the total percentages of HCWs with pre-season HI titres ≤ 40 among infected HCWs. (B) Correlation between infection rates and pre-season HI titres against A/H1N1pdm09. A/H3N2. B/Vic and B/Yam viruses. Symbols show infection rates (% infected/total unvaccinated HCWs) that were calculated for each pre-season HI titre using combined 4-season HI data from paired pre- and post-season serum samples (A/H1N1pdm09 n = 304. A/H3N2 n = 399. B/Vic n = 116. or B/Yam n = 188). Data were fitted with an exponential plateau non-linear regression line with 95% confidence intervals. The estimated values of x at $y_{1/2}$ (50% reduction from the starting y). x at $y_{plateau}$. the rate constant k. the coefficient of variance r^2. and the root mean square error (*rmse*) are reported.

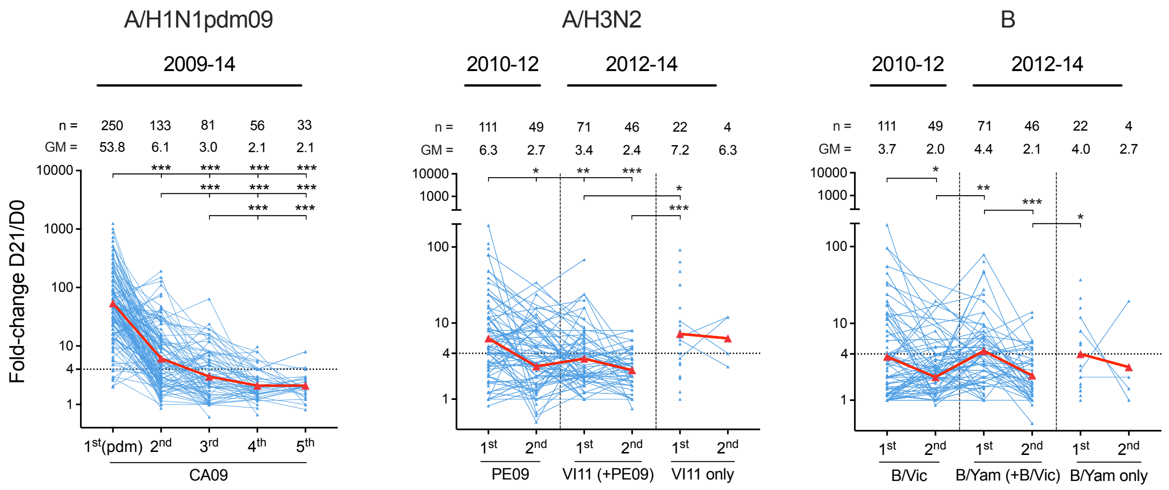

Supplementary Figure 5: The five-year dynamic of hemagglutination-inhibition (HI) antibody fold-change after repeated vaccinations against the same influenza A viruses or B lineages in healthcare workers

All healthcare workers (HCWs) were vaccinated with the AS03-adjuvanted pandemic vaccine containing the A/California/7/2009(H1N1pdm09) (CA09) virus in 2009. During 2010-14. the trivalent seasonal influenza vaccines (TIV) included the same A/H1N1pdm09 CA09 strain. while the A/H3N2 and B components changed from A/Perth/16/2009(H3N2) (PE09) and B/Brisbane/60/2008 of B/Victoria lineage (B/Vic) during 2010-12 to A/Victoria/361/2011(H3N2) (VI11) and B/Wisconsin/1/2010 or B/Massachusetts/2/2012 of B/Yamagata lineage (B/Yam) during 2012-14. The dynamic of fold-changes in HI titres between pre- and 21 days post-vaccination are presented by the sequential order of vaccination against the same influenza A viruses or B lineages. They are designated as 1st. 2nd. 3rd. 4th. and 5th CA09 vaccination for A/H1N1 virus; 1st and 2nd PE09. 1st and 2nd VI11 with previous PE09 vaccination for A/H3N2 viruses; and 1st and 2nd B/VI. 1st and 2nd B/YA with previous B/VI vaccination for B viruses. HCWs who were vaccinated in the last 2 seasons only 2012-14 were included and designated as 1st and 2nd VI11 only or 1st and 2nd B/YA only. Symbols with connecting lines represent an individual's response. The symbols with connecting lines in red indicate the geometric means (GM). The dotted line represents the seroconversion antibody fold-change of 4. The number of HCWs and the geometric means are reported above the graph. The effect of repeated vaccination on log-transformed HI titres were analysed in mixed-effect models. followed by post-hoc tests comparing estimated means between the sequential order of vaccinations with Holm-Sidak's multiple comparison tests. Levels of significance are shown above the graph. *p<0.05. **p<0.01. ***p<0.001.

Supplementary Table 1: Demographics and clinical characteristics of the study cohort.

Characteristics	HCWs (N=250)[1]
**Age (years old)**	
Median (range)	38 (21 - 67)
**Birth year**	
Median (range)	1971 (1942-88)
**Sex**	
Female	195 (78.0%)
Male	55 (22.0%)
**Working department**	
Infectious disease	44 (17.6%)
Clinical	107 (42.8%)
Non-clinical	99 (39.6%)
**High-risk conditions[2]**	26 (10.4%)
**Seasonal influenza vaccination status[3]**	
Before 2009	150 (60.0%)
2009/10	45 (18.0%)
2010/11	93/219 (42.5%)
2011/12	67/190 (35.3%)
2012/13	64/181 (35.4%)
2013/14	79/181 (43.6%)
All 4 seasons (2010-14)	33/181 (18.2%)
No seasonal vaccination (pandemic only)	64/181 (35.4%)

[1]Data are presented as number (%). unless otherwise specified.
[2]High-risk conditions include pregnancy. chronic respiratory diseases. neurological diseases. immunosuppressive diseases. heart diseases. diabetes. and obesity.
[3]Seasonal influenza vaccination status before 2009 and season 2009/10 was obtained through questionnaires.

Supplementary Table 2: Demographics and clinical characteristics of influenza A/H1N1pdm09-infected and uninfected healthcare workers.

Characteristic[1]	2010/11			2012/13			2013/14		
	Infected (n=18)	Uninfected (n=98)	*P*-value[2]	Infected (n=8)	Uninfected (n=91)	*P*-value[2]	Infected (n=6)	Uninfected (n=83)	*P*-value[2]
**Age (years old)**			0.7			0.3			0.8
Median	41.7	41.0		45.1	40.1		41.3	38.8	
Range	25 - 64	22 - 64		25 - 62	22 - 66		32 - 55	24 - 66	
**Birth year**						0.3			0.8
Median	1967	1968	0.7	1964	1969		1968	1970	
Range	1945-84	1945-87		1947-84	1943-87		1954-77	1943-85	
**Sex**			0.2			1.0			0.7
Female	17 (94.4%)	76 (77.6%)		7 (87.5%)	74 (81.3%)		5 (83.3%)	71 (85.5%)	
Male	1 (5.6%)	22 (22.4%)		1 (12.5%)	17 (18.7%)		1 (16.7%)	12 (14.5%)	
**Working department**			0.5			0.5			0.4
Infectious	2 (11.1%)	10 (10.2%)		0 (0.0%)	12 (13.2%)		0 (0.0%)	13 (15.7%)	
Clinical	4 (22.2%)	42 (42.9%)		4 (50.0%)	40 (43.9%)		4 (66.7%)	38 (45.8%)	
Non-clinical	12 (66.7%)	46 (46.9%)		4 (50.0%)	39 (42.9%)		2 (33.3%)	32 (38.5%)	
**High-risk conditions[3]**	3 (16.7%)	8 (8.2%)	0.6	0 (0.0%)	7 (7.7%)	0.8	1 (16.7%)	8 (9.2%)	0.3
**Seasonal vaccination before 2009**	9 (50.0%)	47 (48.0%)	1.0	3 (37.5%)	47 (51.6%)	0.7	1 (16.7%)	40 (48.2%)	0.6
**Seasonal vaccination in 2009/10**	4 (22.2%)	12 (12.2%)	0.3	0 (0.0%)	12 (13.2%)	0.1	0 (0.0%)	10 (12.0%)	0.2
**Pre-season HI titres <40 against A/H1N1pdm09**	13 (72.2%)	22 (29.0%)	**<0.001**	4 (50.0%)	22 (24.2%)	0.1	5 (83.3%)	18 (21.7%)	**0.006**

[1]Data are presented as number (%). unless otherwise specified.

[2]*P*-value was determined by Kruskal-Wallis rank-sum test for age or Pearson's Chi-squared test for other characteristics.

[3]High-risk conditions include pregnancy. chronic respiratory diseases. neurological diseases. immunosuppressive diseases. heart diseases. diabetes. and obesity.

Supplementary Table 3: Demographics and clinical characteristics of influenza A/H3N2-infected and uninfected healthcare workers.

Characteristic[1]	2010/11			2011/12			2012/13			2013/14		
	Infected (n=6)	Uninfected (n=110)	*P*-value[2]	Infected (n=8)	Uninfected (n=87)	*P*-value[2]	Infected (n=8)	Uninfected (n=91)	*P*-value[2]	Infected (n=6)	Uninfected (n=83)	*P*-value[2]
**Age (years old)**			0.5			0.5			0.069			0.2
Median	37.0	41.3		35.1	40.3		48.2	39.8		32.5	39.5	
Range	33 - 49	22 - 64		24 - 46	22 - 62		29 - 62	22 - 66		24 - 54	24 - 66	
**Birth year**			0.5			0.5			0.069			0.2
Median	1972	1968		1974	1969		1961	1969		1976	1969	
Range	1960-76	1945-87		1963-85	1947-87		1947-80	1943-87		1955-85	1943-85	
**Sex**			0.5			0.8			1.0			1.0
Female	6 (100%)	87 (79.1%)		6 (75.0%)	74 (85.1%)		7 (87.5%)	74 (81.3%)		5 (83.3%)	71 (85.5%)	
Male	0 (0%)	23 (20.9%)		2 (25.0%)	13 (14.9%)		1 (12.5%)	17 (18.7%)		1 (16.7%)	12 (14.5%)	
**Working department**			0.2			0.081			0.4			0.3
Infectious	2 (33.3%)	11 (10.0%)		0 (0.0%)	11 (12.6%)		0 (0.0%)	12 (13.2%)		1 (16.7%)	12 (14.5%)	
Clinical	2 (33.3%)	44 (40.0%)		6 (75.0%)	36 (41.4%)		5 (62.5%)	39 (42.9%)		4 (66.6%)	38 (45.8%)	
Non-clinical	2 (33.3%)	55 (50.0%)		2 (25.0%)	34 (39.1%)		3 (37.5%)	40 (43.9%)		1 (16.7%)	33 (39.7%)	
**High-risk conditions[3]**	0 (0.0%)	11 (10.0%)	1.0	1 (12.5%)	6 (6.9%)	1.0	0 (0.0%)	7 (97.7%)	0.8	0 (0.0%)	9 (10.8%)	1.0
**Seasonal vaccination before 2009**	3 (50.0%)	53 (48.2%)	1.0	3 (37.5%)	35 (40.2%)	1.0	5 (62.5%)	45 (49.4%)	0.7	2 (33.3%)	39 (47.0%)	0.5
**Seasonal vaccination in 2009/10**	1 (16.7%)	15 (13.6%)	0.2	0 (0.0%)	9 (10.3%)	0.4	2 (25.0%)	10 (11.0%)	0.3	1 (16.7%)	9 (10.8%)	0.2
**Pre-season HI titres <40 against A/H3N2**	6 (100%)	95 (86.4%)	0.8	8 (100%)	70 (80.5%)	0.4	8 (100%)	83 (91.2%)	0.2	6 (100%)	49 (59.0%)	0.082

[1]Data are presented as number (%). unless otherwise specified.

[2]*P*-value was determined by Kruskal-Wallis rank-sum test for age or Pearson's Chi-squared test for other characteristics.

[3]High-risk conditions include pregnancy. chronic respiratory diseases. neurological diseases. immunosuppressive diseases. heart diseases. diabetes. and obesity.

Supplementary Table 4: Demographics and clinical characteristics of influenza B-infected and uninfected healthcare workers.

Characteristic[1]	2010/11			2012/13			2013/14		
	Infected (n=29)	Uninfected (n=87)	*P*-value[2]	Infected (n=13)	Uninfected (n=86)	*P*-value[2]	Infected (n=16)	Uninfected (n=73)	*P*-value[2]
**Age (years old)**			0.3			**0.037**			0.7
Median	42.8	40.6		45.3	39.7		38.9	39.0	
Range	24 - 64	22 - 64		31 - 61	22 - 66		24 - 66	23 - 61	
**Birth year**			0.3			**0.037**			0.7
Median	1966	1968		1964	1969		1970	1970	
Range	1945-85	1945-87		1948-78	1943-87		1943-85	1948-86	
**Sex**			**0.024**			0.5			1.0
Female	28 (96.6%)	65 (74.7%)		12 (92.3%)	69 (80.2%)		14 (87.5%)	62 (84.9%)	
Male	1 (3.4%)	22 (25.3%)		1 (7.7%)	17 (19.8%)		2 (12.5%)	11 (15.1%)	
**Working department**			1.0			0.11			0.2
Infectious	3 (10.3%)	10 (11.5%)		0 (0%)	12 (14.0%)		2 (12.5%)	11 (15.1%)	
Clinical	13 (44.8%)	33 (37.9%)		5 (38.5%)	39 (45.3%)		5 (31.2%)	37 (50.7%)	
Non-clinical	13 (44.8%)	44 (50.6%)		8 (61.5%)	35 (40.7%)		9 (56.3%)	25 (34.2%)	
**High-risk conditions[3]**	1 (3.4%)	10 (11.5%)	0.4	0 (0.0%)	7 (8.1%)	0.5	1 (6.2%)	8 (11.0%)	0.9
**Seasonal vaccination before 2009**	7 (24.1%)	49 (56.3%)	**0.003**	4 (30.8%)	46 (53.5%)	0.4	5 (31.2%)	36 (49.3%)	0.3
**Seasonal vaccination in 2009/10**	2 (6.9%)	14 (16.1%)	0.5	1 (7.7%)	11 (12.8%)	0.3	2 (12.5%)	8 (11.0%)	0.5
**Pre-season HI titres <40 against B**	25 (86.2%)	38 (43.7%)	**<0.001**	12 (92.3%)	33 (38.4%)	**<0.001**	10 (62.5%)	20 (27.4%)	**0.023**

[1]Data are presented as number (%). unless otherwise specified.
[2]*P*-value was determined by Kruskal-Wallis rank-sum test for age or Pearson's Chi-squared test for other characteristics.
[3]High-risk conditions include pregnancy. chronic respiratory diseases. neurological diseases. immunosuppressive diseases. heart diseases. diabetes. and obesity.

Supplementary Table 5: Demographics and clinical characteristics of healthcare workers in season 2010/11.

Healthcare workers were divided into four groups: vaccinated with the trivalent seasonal influenza vaccines (TIV) in both 2009/10 and 2010/11 (Previous & current). vaccinated in the current season 2010/11 only (Current only). vaccinated in the previous season 2009/10 only (Previous only). and unvaccinated in both seasons (Unvaccinated). The seasonal vaccination status in 2009 was obtained through questionnaires.

**Characteristic**[1]	**Previous & current (n=23)**	**Current only (n=70)**	**Previous only (n=17)**	**Unvaccinated (n=109)**	***P*-value**[2]
**Age (years old)**					0.790
Median	36	40	41	39	
Range	26 - 64	22 - 67	21 - 63	22 - 66	
**Birth year**					0.790
Median	1973	1969	1968	1970	
Range	1945-83	1942-87	1946-88	1943-87	
**Sex**					0.740
Female	17 (73.9%)	52 (74.3%)	13 (76.5%)	88 (80.7%)	
Male	6 (26.1%)	18 (25.7%)	4 (23.5%)	21 (19.3%)	
**Working department**					**<0.001**
Infectious	13 (56.5%)	15 (21.4%)	5 (29.4%)	9 (8.3%)	
Clinical	2 (8.7%)	32 (45.7%)	1 (5.9%)	54 (49.5%)	
Non-clinical	8 (34.8%)	23 (32.9%)	11 (64.7%)	46 (42.2%)	
**High-risk conditions**[3]	1 (4.3%)	9 (12.9%)	3 (17.6%)	9 (8.3%)	0.410
**Seasonal vaccination before 2009**	18 (78.3%)	52 (74.3%)	11 (64.7%)	52 (47.7%)	**0.001**
**Pre-season HI titres <40 against A/H1N1pdm09**	9 (39.1%)	25 (35.7%)	3/16 (18.7%)	22/100 (22.1%)	0.140
**Pre-season HI titres <40 against A/H3N2**	14 (60.9%)	53 (75.7%)	10/16 (62.5%)	91/100 (91.0%)	**<0.001**
**Pre-season HI titres <40 against B/Vic**	3 (13.0%)	19 (27.1%)	4/16 (25.0%)	47/100 (47.0%)	**0.006**

[1]Data are presented as number (%). unless otherwise specified.

[2]*P*-value was determined by Kruskal-Wallis rank-sum test for age or Pearson's Chi-squared test for other characteristics.

[3]High-risk conditions include pregnancy. chronic respiratory diseases. neurological diseases. immunosuppressive diseases. heart diseases. diabetes. and obesity.

Supplementary Table 6: Demographics and clinical characteristics of healthcare workers in season 2011/12.

Healthcare workers were divided into four groups: vaccinated with the trivalent seasonal influenza vaccines (TIV) in both 2010/11 and 2011/12 (Previous & current). vaccinated in the current season 2011/12 only (Current only). vaccinated in the previous season 2010/11 only (Previous only). and unvaccinated in both seasons (Unvaccinated).

Characteristic[1]	Previous & current (n=49)	Current only (n=18)	Previous only (n=29)	Unvaccinated (n=94)	*P*-value[2]
**Age (years old)**					0.064
Median	41	46	33	40	
Mix - Max	26 - 67	22 - 63	22 - 62	23 - 64	
**Birth year**					0.064
Median	1968	1963	1976	1969	
Range	1942-83	1946-87	1947-87	1945-86	
**Sex**					0.720
Female	39 (79.6%)	13 (72.2%)	23 (79.3%)	79 (84.0%)	
Male	10 (20.4%)	5 (27.8%)	6 (20.7%)	15 (16.0%)	
**Working department**					**<0.001**
Infectious	18 (36.7%)	5 (27.8%)	7 (24.1%)	7 (7.4%)	
Clinical	14 (28.6%)	4 (22.2%)	16 (55.2%)	48 (51.1%)	
Non-clinical	17 (34.7%)	9 (50.0%)	6 (20.7%)	39 (41.5%)	
**High-risk conditions[3]**	3 (6.1%)	2 (11.1%)	4 (13.8%)	8 (8.5%)	0.520
**Seasonal vaccination before 2009**	44 (89.8%)	16 (88.9%)	15 (51.7%)	40 (42.6%)	**<0.001**
**Seasonal vaccination in 2009/10**	13 (26.5%)	6 (33.3%)	4 (13.8%)	8 (8.5%)	**0.016**
**Pre-season HI titres <40 against A/H1N1pdm09**	13 (36.5%)	2 (11.1%)	5/28 (17.9%)	20/92 (21.7%)	0.570
**Pre-season HI titres <40 against A/H3N2**	26 (55.1%)	16 (88.9%)	13/28 (46.4%)	83/92 (90.2%)	**<0.001**
**Pre-season HI titres <40 against B/Vic**	8 (16.3%)	8 (44.4%)	3/28 (10.7%)	27/92 (29.3%)	**0.025**

[1]Data are presented as number (%). unless otherwise specified.

[2]*P*-value was determined by Kruskal-Wallis rank-sum test for age or Pearson's Chi-squared test for other characteristics.

[3]High-risk conditions include pregnancy. chronic respiratory diseases. neurological diseases. immunosuppressive diseases. heart diseases. diabetes. and obesity.

Supplementary Table 7: Demographics and clinical characteristics of healthcare workers in season 2012/13.

Healthcare workers were divided into four groups: vaccinated with the trivalent seasonal influenza vaccines (TIV) in both 2011/12 and 2012/13 (Previous & current). vaccinated in the current season 2012/13 only (Current only). vaccinated in the revious season 2011/12 only (Previous only). and unvaccinated in both seasons (Unvaccinated).

Characteristic[1]	Previous & current (n=47)	Current only (n=17)	Previous only (n=17)	Unvaccinated (n=100)	*P*-value[2]
**Age (years old)**					0.190
Median	44	43	49	37	
Mix - Max	22 - 67	23 - 58	24 - 62	22 - 66	
**Birth year**					0.190
Median	1965	1966	1960	1972	
Range	1942-87	1951-86	1947-85	1943-87	
**Sex**					0.800
Female	37 (78.7%)	15 (88.2%)	13 (76.5%)	83 (83.0%)	
Male	10 (21.3%)	2 (11.8%)	4 (23.5%)	17 (17.0%)	
**Working department**					**<0.001**
Infectious	18 (38.3%)	1 (5.9%)	4 (23.5%)	13 (13.0%)	
Clinical	11 (23.4%)	15 (88.2%)	7 (41.2%)	48 (48.0%)	
Non-clinical	18 (38.3%)	1 (5.9%)	6 (35.3%)	39 (39.0%)	
**High-risk conditions[3]**	4 (8.5%)	3 (17.6%)	1 (5.9%)	9 (9.0%)	0.590
**Seasonal vaccination before 2009**	42 (89.4%)	10 (58.8%)	16 (94.1%)	42 (42.0%)	**<0.001**
**Seasonal vaccination in 2009/10**	12 (25.5%)	0 (0.0%)	5 (29.4%)	10 (10.0%)	**0.011**
**Pre-season HI titres <40 against A/H1N1pdm09**	4 (8.5%)	6 (35.3%)	2/16 (12.5%)	28/96 (29.2%)	0.051
**Pre-season HI titres <40 against A/H3N2**	23 (48.9%)	14 (82.4%)	6/16 (37.5%)	70/96 (72.9%)	**0.017**
**Pre-season HI titres <40 against B/Yam**	21 (44.7%)	7 (41.2%)	7/16 (43.7%)	49/96 (51.0%)	0.950

[1]Data are presented as number (%). unless otherwise specified.
[2]*P*-value was determined by Kruskal-Wallis rank-sum test for age or Pearson's Chi-squared test for other characteristics.
[3]High-risk conditions include pregnancy. chronic respiratory diseases. neurological diseases. immunosuppressive diseases. heart diseases. diabetes. and obesity.

Supplementary Table 8: Demographics and clinical characteristics of healthcare workers in season 2013/14.

Healthcare workers (HCW) were divided into four groups: vaccinated with the trivalent seasonal influenza vaccines (TIV) in both 2012/13 and 2013/14 (Previous & current). vaccinated in the current season 2013/14 only (Current only). vaccinated in the previous season 2012/13 only (Previous only). and unvaccinated in both seasons (Unvaccinated).

Characteristic[1]	Previous & current (n=50)	Current only (n=29)	Previous only (n=14)	Unvaccinated (n=88)	*P*-value[2]
**Age (years old)**					0.095
Median	45	44	36	37	
Mix - Max	22 - 67	22 - 62	23 - 55	24 - 66	
**Birth year**					0.095
Median	1964	1965	1973	1972	
Range	1942-87	1947-87	1954-86	1943-85	
**Sex**					0.480
Female	40 (80.0%)	23 (79.3%)	13 (92.9%)	72 (81.8%)	
Male	10 (20.0%)	6 (20.7%)	1 (7.1%)	16 (18.2%)	
**Working department**					0.084
Infectious	16 (32.0%)	5 (17.2%)	4 (28.6%)	11 (12.5%)	
Clinical	18 (36.0%)	14 (48.3%)	7 (50.0%)	42 (47.7%)	
Non-clinical	16 (32.0%)	10 (34.5%)	3 (21.4%)	35 (39.8%)	
**High-risk conditions[3]**	5 (10.0%)	2 (6.9%)	2 (14.3%)	8 (9.1%)	0.640
**Seasonal vaccination before 2009**	43 (86.0%)	20 (69.0%)	9 (64.3%)	38 (43.2%)	**<0.001**
**Seasonal vaccination in 2009/10**	11 (22.0%)	4 (13.8%)	2 (14.3%)	11 (12.5%)	0.430
**Pre-season HI titres <40 against A/H1N1pdm09**	10 (20.0%)	4 (13.8%)	2/12 (16.7%)	27/82 (32.9%)	0.100
**Pre-season HI titres <40 against A/H3N2**	21 (42.0%)	15 (51.7%)	4/12 (33.3%)	56/82 (68.3%)	**0.049**
**Pre-season HI titres <40 against B/Yam**	5 (10.0%)	3 (10.3%)	2/12 (16.7%)	30/82 (36.6%)	**0.010**

[1]Data are presented as number (%). unless otherwise specified.
[2]*P*-value was determined by Kruskal-Wallis rank-sum test for age or Pearson's Chi-squared test for other characteristics.
[3]High-risk conditions include pregnancy. chronic respiratory diseases. neurological diseases. immunosuppressive diseases. heart diseases. diabetes. and obesity.

Supplementary Table 9: Statistical analysis for Figure 3B.

Analysis			Adjusted p value	t value	Degree of freedom	Summary
**A/H1N1pdm09**						
	CA09 Infection (n = 31)					
		Pre vs. Post	**<0.001**	12.01	30.00	***
		Pre vs. 12M	**<0.001**	7.063	21.00	***
		Pre vs. 24M	**0.004**	4.502	8.000	**
		Pre vs. 36M	0.885	0.151	6.000	ns
	CA09 First TIV (n = 51)					
		Pre vs. Post	**<0.001**	5.006	50.00	***
		Pre vs. 12M	**0.002**	3.933	25.00	**
		Pre vs. 24M	0.1997	1.751	12.00	ns
		Pre vs. 36M	0.4792	0.764	5.000	ns
	CA09 Infection (n = 31) vs. CA09 First TIV (n = 51)					
		Pre	**<0.001**	4.186	68.68	###
		Post	0.642	0.468	59.42	ns
		12M	0.241	1.547	45.95	ns
		24M	**0.026**	2.922	19.11	#
		36M	**0.024**	3.599	8.741	#
**A/H3N2**						
	PE09 Infection (n = 14)					
		Pre vs. Post	**<0.001**	10.95	13.00	***
		Pre vs. 12M	**0.002**	4.271	9.000	**
	PE09 First TIV (n = 34)					
		Pre vs. Post	**<0.001**	7.198	33.00	***
		Pre vs. 12M	**<0.001**	4.504	15.00	***
	PE09 Infection (n = 14) vs. PE09 First TIV (n = 34)					
		Pre	0.171	1.788	19.27	ns
		Post	**0.001**	3.804	38.13	##
		12M	0.260	1.161	19.02	ns
	VI11 Infection (n = 14)					
		Pre vs. Post	**<0.001**	13.49	13.00	***
		Pre vs. 12M	**0.004**	6.093	4.000	**
	VI11 First TIV (n = 17)					
		Pre vs. Post	**0.005**	3.622	16.00	**
		Pre vs. 12M	0.109	2.058	4.000	ns
	VI11 Infection (n = 14) vs. VI11 First TIV (n = 17)					
		Pre	0.727	0.353	28.03	ns
		Post	**0.002**	3.814	27.74	##
		12M	0.466	1.266	4.368	ns
**B**						
	B/Vic Infection (n = 29)					
		Pre vs. Post	**<0.001**	17.42	28.00	***
		Pre vs. 12M	**0.002**	3.550	19.00	**
	B/Vic First TIV (n = 34)					
		Pre vs. Post	**<0.001**	5.727	33.00	***
		Pre vs. 12M	**0.029**	2.421	15.00	*

Analysis			Adjusted p value	t value	Degree of freedom	Summary
	B/Vic Infection (n = 29) vs. B/Vic First TIV (n = 34)					
		Pre	**0.005**	3.285	60.99	##
		Post	0.823	0.225	60.86	ns
		12M	**0.048**	2.289	33.77	#
	B/Yam Infection (n = 26)					
		Pre vs. Post	**<0.001**	18.89	25.00	***
		Pre vs. 12M	**0.018**	3.236	6.000	*
	B/Yam First TIV (n = 17)					
		Pre vs. Post	0.137	1.932	16.00	ns
		Pre vs. 12M	0.410	0.899	5.000	ns
	B/Yam Infection (n = 26) vs. B/Yam First TIV (n = 17)					
		Pre	**0.002**	3.887	29.73	##
		Post	0.798	0.258	29.85	ns
		12M	0.392	1.300	10.96	ns

Data was log-transformed and analysed using mixed-effects model with the Geisser-Greenhouse correction and Holm-Sidak's multiple comparisons test with individual variances computed for each comparison.
ns – not significant p value > 0.05

Supplementary Table 10: Statistical analysis for Figure 3C

Analysis			Adjusted p value	t value	Degree of freedom	Summary
**A/H1N1pdm09**						
	Hybrid (n = 5. A/H1N1pdm09 infected + subsequent TIV)					
		D0 vs. D21	**<0.001**	6.892	63.00	***
		D0 vs. 3M	**<0.001**	4.792	63.00	***
		D0 vs. 6M	**0.012**	2.829	63.00	*
		D0 vs. 12M	0.282	1.085	63.00	ns
	Second TIV (n = 16)					
		D0 vs. D21	**<0.001**	4.644	63.00	***
		D0 vs. 3M	**0.031**	2.644	63.00	*
		D0 vs. 6M	0.138	1.832	63.00	ns
		D0 vs. 12M	0.508	0.665	63.00	ns
**A/H3N2**						
	Hybrid (n = 6. A/H3N2 infected + subsequent TIV)					
		D0 vs. D21	**0.001**	3.852	75.00	***
		D0 vs. 3M	0.056	2.393	75.00	ns
		D0 vs. 6M	0.168	1.728	75.00	ns
		D0 vs. 12M	0.923	0.097	75.00	ns
	Second TIV (n = 16)					
		D0 vs. D21	**<0.001**	10.21	75.00	***
		D0 vs. 3M	**<0.001**	8.157	75.00	***
		D0 vs. 6M	**<0.001**	6.585	75.00	***
		D0 vs. 12M	**<0.001**	3.837	75.00	***
	Hybrid (n = 6) vs. Second TIV (n = 16)					
		Pre	**0.014**	2.477	135.0	#
		Post	**<0.001**	5.771	135.0	###
		D0	**<0.001**	4.802	135.0	###
		D21	**0.014**	2.870	135.0	#
		3M	**0.014**	2.774	135.0	#
		6M	**0.011**	3.096	135.0	#
		12M	**0.011**	3.110	135.0	#
**B**						
	Hybrid (n = 6. B infected + subsequent TIV)					
		D0 vs. D21	**0.001**	3.776	71.00	**
		D0 vs. 3M	**0.010**	3.025	71.00	*
		D0 vs. 6M	**0.017**	2.692	71.00	*
		D0 vs. 12M	0.113	1.606	71.00	ns
	Second TIV (n = 16)					
		D0 vs. D21	**<0.001**	6.104	71.00	***
		D0 vs. 3M	**<0.001**	4.611	71.00	***
		D0 vs. 6M	**<0.001**	4.102	71.00	***
		D0 vs. 12M	**0.020**	2.379	71.00	*

Data was log-transformed and analysed using mixed-effects model and Holm-Sidak's multiple comparisons test with a single pooled variance.
ns – not significant p value > 0.05

Supplementary Table 11: Statistical analysis for Figure 4.

Analysis				Adjusted p value	t value	Degree of freedom	Summary
**Fig. 4A – Season 2010/11**							
	A/H1N1pdm09						
		Current & Previous (n = 23)	D0 vs. D21	**<0.001**	7.289	21.00	***
			D0 vs. 3M	**<0.001**	6.708	21.00	***
			D0 vs. 6M	**<0.001**	5.053	21.00	***
			D0 vs. 12M	**0.048**	2.102	21.00	*
		Current Only (n = 70)	D0 vs. D21	**<0.001**	12.99	67.00	***
			D0 vs. 3M	**<0.001**	9.935	64.00	***
			D0 vs. 6M	**<0.001**	7.712	65.00	***
			D0 vs. 12M	**<0.001**	5.093	63.00	***
		Previous Only (n = 17)	D0 vs. 12M	0.993	0.009	10.00	ns
		Unvaccinated (n = 109)	D0 vs. 12M	0.090	1.715	93.00	ns
	**A/H3N2**						
		Current & Previous (n = 23)	D0 vs. D21	**<0.001**	4.665	21.00	***
			D0 vs. 3M	**0.008**	3.426	21.00	**
			D0 vs. 6M	**0.016**	2.917	22.00	*
			D0 vs. 12M	0.152	1.485	22.00	ns
		Current Only (n = 70)	D0 vs. D21	**<0.001**	14.27	68.00	***
			D0 vs. 3M	**<0.001**	11.31	65.00	***
			D0 vs. 6M	**<0.001**	9.357	66.00	***
			D0 vs. 12M	**<0.001**	7.055	65.00	***
		Previous Only (n = 17)	D0 vs. 12M	0.126	1.622	15.00	ns
		Unvaccinated (n = 109)	D0 vs. 12M	**0.044**	2.036	98.00	*
		Current & Previous vs. Unvaccinated	D0	**0.004**	3.595	28.81	**
			12M	**<0.001**	5.147	27.93	***
		Current Only vs. Unvaccinated	D0	**0.023**	2.553	130.8	*
			12M	**<0.001**	8.826	104.1	***
		Previous Only vs. Unvaccinated	D0	**0.023**	2.713	17.09	*
			12M	**0.025**	2.469	16.75	*
	**B**						
		Current & Previous (n = 23)	D0 vs. D21	**<0.001**	5.391	22.00	***
			D0 vs. 3M	**<0.001**	4.752	22.00	***
			D0 vs. 6M	**0.003**	3.691	21.00	**
			D0 vs. 12M	0.095	1.745	21.00	ns
		Current Only (n = 70)	D0 vs. D21	**<0.001**	8.712	67.00	***
			D0 vs. 3M	**<0.001**	7.571	65.00	***
			D0 vs. 6M	**<0.001**	6.484	65.00	***
			D0 vs. 12M	**<0.001**	4.615	65.00	***
		Previous Only (n = 17)	D0 vs. 12M	0.078	1.904	14.00	ns
		Unvaccinated (n = 109)	D0 vs. 12M	0.977	0.029	76.00	ns
		Current & Previous vs. Unvaccinated	D0	**0.011**	3.107	36.81	*
			12M	**<0.001**	4.430	40.21	***
		Current Only vs. Unvaccinated	D0	**0.028**	2.483	141.3	*
			12M	**<0.001**	5.894	140.9	***
		Previous Only vs. Unvaccinated	D0	**0.030**	2.358	17.80	*
			12M	0.094	1.768	17.62	ns

Analysis				Adjusted p value	t value	Degree of freedom	Summary
**Fig. 4B – Season 2011/12**							
	**A/H1N1pdm09**						
		Current & Previous (n = 49)	D0 vs. D21	**<0.001**	9.292	44.00	***
			D0 vs. 3M	**<0.001**	6.969	44.00	***
			D0 vs. 6M	**<0.001**	5.610	43.00	***
			D0 vs. 12M	**0.003**	3.093	47.00	**
		Current Only (n = 18)	D0 vs. D21	**<0.001**	6.690	16.00	***
			D0 vs. 3M	**<0.001**	5.899	16.00	***
			D0 vs. 6M	**<0.001**	5.068	16.00	***
			D0 vs. 12M	**<0.001**	4.271	17.00	***
		Previous Only (n = 29)	D0 vs. 12M	**0.018**	2.537	25.00	*
		Unvaccinated (n = 94)	D0 vs. 12M	**<0.001**	11.00	78.00	***
		Current & Previous vs. Current Only	D0	0.944	0.216	39.50	ns
			D21	0.066	1.909	29.37	ns
			3M	**0.017**	2.519	34.60	#
			6M	**0.021**	2.400	41.04	#
			12M	**0.019**	3.033	44.88	#
		Current & Previous vs. Unvaccinated	D0	0.894	0.634	98.25	ns
			12M	0.150	2.072	104.8	ns
		Current Only vs. Unvaccinated	D0	0.944	0.303	30.34	ns
			12M	**<0.001**	5.199	38.84	###
		Previous Only vs. Unvaccinated	D0	0.619	1.357	42.68	ns
			12M	0.345	1.333	37.40	ns
	**A/H3N2**						
		Current & Previous (n = 49)	D0 vs. D21	**<0.001**	6.638	44.00	***
			D0 vs. 3M	**<0.001**	4.993	44.00	***
			D0 vs. 6M	**0.010**	2.953	43.00	*
			D0 vs. 12M	0.212	1.265	46.00	ns
		Current Only (n = 18)	D0 vs. D21	**<0.001**	6.996	15.00	***
			D0 vs. 3M	**<0.001**	5.057	15.00	***
			D0 vs. 6M	**0.001**	4.367	15.00	**
			D0 vs. 12M	**0.006**	3.198	16.00	**
		Previous Only (n = 29)	D0 vs. 12M	**0.009**	2.848	23.00	**
		Unvaccinated (n = 94)	D0 vs. 12M	0.309	1.025	69.00	ns
		Current & Previous vs. Unvaccinated	D0	**<0.001**	5.435	94.83	###
			12M	**<0.001**	6.892	102.0	###
		Current Only vs. Unvaccinated	D0	0.358	0.940	20.01	ns
			12M	**<0.001**	4.606	23.38	###
		Previous Only vs. Unvaccinated	D0	**<0.001**	6.069	42.43	###
			12M	**<0.001**	4.600	33.01	###
	**B**						
		Current & Previous (n = 49)	D0 vs. D21	**<0.001**	7.828	44.00	***
			D0 vs. 3M	**<0.001**	6.119	44.00	***
			D0 vs. 6M	**<0.001**	4.873	43.00	***
			D0 vs. 12M	**0.046**	2.049	46.00	*
		Current Only (n = 18)	D0 vs. D21	**<0.001**	5.034	15.00	***
			D0 vs. 3M	**0.002**	4.394	15.00	**
			D0 vs. 6M	**0.006**	3.568	15.00	**

Analysis				Adjusted p value	t value	Degree of freedom	Summary
			D0 vs. 12M	0.094	1.782	16.00	ns
		Previous Only (n = 29)	D0 vs. 12M	**<0.001**	9.201	17.00	***
		Unvaccinated (n = 94)	D0 vs. 12M	**<0.001**	7.355	72.00	***
		Current & Previous	D0	**0.006**	3.010	113.8	##
		vs. Unvaccinated	12M	**<0.001**	4.583	108.2	###
		Current Only	D0	0.654	0.4545	22.65	ns
		vs. Unvaccinated	12M	**0.023**	2.413	26.15	#
		Previous Only	D0	**<0.001**	4.272	50.21	###
		vs. Unvaccinated	12M	**0.016**	2.858	27.61	#
**Fig. 4C – Season 2012/13**							
	**A/H1N1pdm09**						
		Current & Previous	D0 vs. D21	**<0.001**	8.954	45.00	***
		(n = 47)	D0 vs. 3M	**<0.001**	6.456	44.00	***
			D0 vs. 6M	**0.016**	2.510	44.00	*
			D0 vs. 12M	**0.001**	3.679	44.00	**
		Current Only (n = 17)	D0 vs. D21	**<0.001**	5.808	14.00	***
			D0 vs. 3M	**0.002**	4.449	14.00	**
			D0 vs. 6M	**0.007**	3.617	12.00	**
			D0 vs. 12M	0.065	2.012	13.00	ns
		Previous Only (n = 17)	D0 vs. 12M	**0.044**	2.202	15.00	*
		Unvaccinated (n = 100)	D0 vs. 12M	**<0.001**	6.610	77.00	***
	**A/H3N2**						
		Current & Previous	D0 vs. D21	**<0.001**	10.46	45.00	***
		(n = 47)	D0 vs. 3M	**<0.001**	6.960	44.00	***
			D0 vs. 6M	**<0.001**	5.258	44.00	***
			D0 vs. 12M	0.059	1.935	43.00	ns
		Current Only (n = 17)	D0 vs. D21	**<0.001**	6.922	14.00	***
			D0 vs. 3M	**<0.001**	5.743	14.00	***
			D0 vs. 6M	**0.002**	4.327	12.00	**
			D0 vs. 12M	**0.025**	2.529	13.00	*
		Previous Only (n = 17)	D0 vs. 12M	0.311	1.047	15.00	ns
		Unvaccinated (n = 100)	D0 vs. 12M	**<0.001**	3.940	76.00	***
		Current & Previous	D0	**0.003**	3.367	111.8	##
		vs. Unvaccinated	12M	**<0.001**	5.283	121.9	###
		Current Only	D0	0.822	0.227	21.61	ns
		vs. Unvaccinated	12M	0.161	1.455	20.40	ns
		Previous Only	D0	**0.047**	2.421	22.44	#
		vs. Unvaccinated	12M	**0.039**	2.506	23.55	#
	**B**						
		Current & Previous	D0 vs. D21	**<0.001**	11.40	46.00	***
		(n = 47)	D0 vs. 3M	**<0.001**	8.693	45.00	***
			D0 vs. 6M	**<0.001**	6.956	45.00	***
			D0 vs. 12M	**<0.001**	4.829	45.00	***
		Current Only (n = 17)	D0 vs. D21	**<0.001**	5.573	11.00	***
			D0 vs. 3M	**0.002**	4.716	11.00	**
			D0 vs. 6M	**0.005**	4.066	10.00	**
			D0 vs. 12M	**0.013**	3.025	10.00	*
		Previous Only (n = 17)	D0 vs. 12M	0.071	1.950	14.00	ns

Analysis				Adjusted p value	t value	Degree of freedom	Summary
		Unvaccinated (n = 100)	D0 vs. 12M	0.241	1.182	72.00	ns
		Current & Previous vs. Unvaccinated	D0	0.883	0.445	109.7	ns
			12M	**0.008**	3.074	114.1	##
		Current Only vs. Unvaccinated	D0	0.307	1.642	21.75	ns
			12M	**0.009**	3.066	31.87	##
		Previous Only vs. Unvaccinated	D0	0.883	0.404	21.34	ns
			12M	0.669	0.434	21.99	ns
**Fig. 4D – Season 2013/14**							
	**A/H1N1pdm09**						
		Current & Previous (n = 50)	D0 vs. D21	**<0.001**	9.417	48.00	***
			D0 vs. 3M	**<0.001**	4.648	48.00	***
			D0 vs. 6M	**0.013**	2.600	48.00	*
			D0 vs. 12M	**0.013**	2.843	48.00	*
		Current Only (n = 29)	D0 vs. D21	**<0.001**	4.522	27.00	***
			D0 vs. 3M	**0.004**	3.600	28.00	**
			D0 vs. 6M	**0.017**	2.819	28.00	*
			D0 vs. 12M	0.948	0.0659	27.00	ns
		Previous Only (n = 14)	D0 vs. 12M	**<0.001**	8.329	8.000	***
		Unvaccinated (n = 88)	D0 vs. 12M	**<0.001**	7.113	77.00	***
		Current & Previous vs. Current Only	D0	0.958	0.2157	39.50	ns
			D21	**0.022**	1.909	29.37	#
			3M	**0.007**	2.519	34.60	##
			6M	**0.018**	2.400	41.04	#
			12M	0.561	3.033	44.88	ns
		Current & Previous vs. Unvaccinated	D0	0.958	0.6341	98.25	ns
			12M	0.258	2.072	104.8	ns
		Current Only vs. Unvaccinated	D0	0.958	0.3033	30.34	ns
			12M	**0.040**	5.199	38.84	#
		Previous Only vs. Unvaccinated	D0	0.101	1.357	42.68	ns
			12M	0.118	1.333	37.40	ns
	**A/H3N2**						
		Current & Previous (n = 50)	D0 vs. D21	**<0.001**	10.67	48.00	***
			D0 vs. 3M	**<0.001**	9.837	48.00	***
			D0 vs. 6M	**<0.001**	7.264	48.00	***
			D0 vs. 12M	**0.046**	2.052	47.00	*
		Current Only (n = 29)	D0 vs. D21	**<0.001**	6.758	29.00	***
			D0 vs. 3M	**<0.001**	6.410	29.00	***
			D0 vs. 6M	**<0.001**	5.426	29.00	***
			D0 vs. 12M	**0.007**	2.888	28.00	**
		Previous Only (n = 14)	D0 vs. 12M	**0.025**	2.760	8.000	*
		Unvaccinated (n = 88)	D0 vs. 12M	0.435	0.7857	73.00	ns
		Current & Previous vs. Unvaccinated	D0	**0.002**	3.551	123.8	##
			12M	**<0.001**	4.571	112.9	###
		Current Only vs. Unvaccinated	D0	0.177	1.369	53.33	ns
			12M	**0.003**	3.349	49.81	##
		Previous Only vs. Unvaccinated	D0	0.085	2.314	9.834	ns
			12M	0.121	1.700	9.587	ns

Analysis				Adjusted p value	t value	Degree of freedom	Summary
	**B**						
		Current & Previous (n = 50)	D0 vs. D21	**<0.001**	6.891	48.00	***
			D0 vs. 3M	**<0.001**	4.050	48.00	***
			D0 vs. 6M	0.117	1.924	48.00	ns
			D0 vs. 12M	0.513	0.6595	48.00	ns
		Current Only (n = 29)	D0 vs. D21	**<0.001**	5.632	28.00	***
			D0 vs. 3M	**<0.001**	4.899	28.00	***
			D0 vs. 6M	**0.002**	3.788	28.00	**
			D0 vs. 12M	**0.023**	2.413	28.00	*
		Previous Only (n = 14)	D0 vs. 12M	**0.013**	3.080	9.000	*
		Unvaccinated (n = 88)	D0 vs. 12M	**0.007**	2.790	65.00	**
		Current & Previous vs. Current Only	D0	0.810	0.5795	55.48	ns
			D21	**0.002**	3.218	67.44	##
			3M	**0.001**	3.451	70.83	###
			6M	**0.013**	2.552	66.48	#
			12M	0.107	2.249	63.19	ns
		Current & Previous vs. Unvaccinated	D0	**0.036**	2.758	112.2	#
			12M	**0.007**	3.293	111.9	##
		Current Only vs. Unvaccinated	D0	**0.036**	2.846	58.06	#
			12M	**<0.001**	5.246	67.55	###
		Previous Only vs. Unvaccinated	D0	0.078	2.645	13.03	ns
			12M	0.116	2.283	12.85	ns

Data was log-transformed and analysed using mixed-effects model with the Geisser-Greenhouse correction and Holm-Sidak's multiple comparisons test with individual variances computed for each comparison.
ns – not significant p value > 0.05

Supplementary Table 12: Statistical analysis for Figure 5.

Analysis		Adjusted p value	t value	Degree of freedom	Summary
**Fig. 5B – Season 2012/13**					
A/H1N1pdm09					
Repeated (n = 33)	D0 vs. D21	**<0.001**	8.875	31.00	***
	D0 vs. 3M	**<0.001**	6.242	31.00	***
	D0 vs. 6M	**0.022**	2.415	32.00	*
	D0 vs. 12M	**0.019**	2.761	32.00	*
First TIV 2012 (n = 11)	D0 vs. D21	**0.002**	5.114	10.00	**
	D0 vs. 3M	**0.006**	4.181	10.00	**
	D0 vs. 6M	**0.026**	3.180	9.000	*
	D0 vs. 12M	0.219	1.334	9.000	ns
Pre-2010 Only (n = 27)	D0 vs. 12M	**0.006**	3.111	20.00	**
No TIV (n = 39)	D0 vs. 12M	**<0.001**	5.527	28.00	***
Repeated vs. First TIV 2012	D0	0.540	0.727	17.88	ns
	D21	0.204	2.067	15.91	ns
	3M	0.166	2.303	15.30	ns
	6M	0.254	1.816	12.78	ns
	12M	0.540	1.034	11.87	ns
A/H3N2					
Repeated (n = 33)	D0 vs. D21	**<0.001**	7.993	31.00	***
	D0 vs. 3M	**<0.001**	4.958	31.00	***
	D0 vs. 6M	**0.002**	3.573	31.00	**
	D0 vs. 12M	0.359	0.932	31.00	ns
First TIV 2012 (n = 11)	D0 vs. D21	**<0.001**	6.491	10.00	***
	D0 vs. 3M	**0.003**	4.903	10.00	**
	D0 vs. 6M	**0.016**	3.679	9.000	*
	D0 vs. 12M	0.053	2.322	9.000	ns
Pre-2010 Only (n = 27)	D0 vs. 12M	0.066	1.942	20.00	ns
No TIV (n = 39)	D0 vs. 12M	**0.013**	2.711	25.00	*
Repeated vs. First TIV 2012	D0	0.059	2.889	13.81	ns
	D21	0.875	0.472	10.46	ns
	3M	0.875	0.393	10.08	ns
	6M	0.478	1.414	8.093	ns
	12M	0.444	1.648	8.319	ns
Repeated vs. No TIV	D0	**<0.001**	4.396	52.44	###
	12M	**<0.001**	7.279	61.40	###
First TIV 2012 vs. No TIV	D0	0.977	0.029	14.72	ns
	12M	0.157	1.548	8.785	ns
Pre-2010 Only vs. No TIV	D0	0.086	2.086	37.06	ns
	12M	**0.019**	2.756	32.15	#
B					
Repeated (n = 33)	D0 vs. D21	**<0.001**	9.931	30.00	***
	D0 vs. 3M	**<0.001**	6.746	30.00	***
	D0 vs. 6M	**<0.001**	5.212	30.00	***
	D0 vs. 12M	**0.002**	3.390	30.00	**
First TIV 2012 (n = 11)	D0 vs. D21	**<0.001**	5.590	10.00	***
	D0 vs. 3M	**0.004**	4.362	10.00	**

Analysis				Adjusted p value	t value	Degree of freedom	Summary
			D0 vs. 6M	**0.010**	3.686	9.000	*
			D0 vs. 12M	**0.020**	2.834	9.000	*
		Pre-2010 Only (n = 27)	D0 vs. 12M	0.567	0.582	20.00	ns
		No TIV (n = 39)	D0 vs. 12M	0.118	1.618	25.00	ns
		Repeated vs. No TIV	D0	**<0.001**	3.871	63.76	###
			12M	**<0.001**	4.596	49.31	###
		First TIV 2012 vs. No TIV	D0	0.063	1.926	31.47	ns
			12M	**0.005**	3.486	18.51	##
		Pre-2010 Only vs. No TIV	D0	**0.001**	3.772	37.14	##
			12M	**0.005**	3.065	40.48	##
**Fig. 5C – Season 2013/14**							
	A/H1N1pdm09						
		Repeated (n = 33)	D0 vs. D21	**<0.001**	8.419	30.00	***
			D0 vs. 3M	**<0.001**	4.831	31.00	***
			D0 vs. 6M	**0.042**	2.425	31.00	*
			D0 vs. 12M	0.148	1.484	31.00	ns
		First TIV 2013 (n = 11)	D0 vs. D21	**0.038**	3.187	10.00	*
			D0 vs. 3M	0.075	2.616	10.00	ns
			D0 vs. 6M	0.111	2.150	10.00	ns
			D0 vs. 12M	0.533	0.645	10.00	ns
		Pre-2010 Only (n = 27)	D0 vs. 12M	**<0.001**	4.489	20.00	***
		No TIV (n = 39)	D0 vs. 12M	**<0.001**	6.171	28.00	***
		Repeated vs. First TIV 2013	D0	0.940	0.077	17.22	ns
			D21	0.088	2.324	20.75	ns
			3M	0.066	2.675	22.86	ns
			6M	0.066	2.604	24.87	ns
			12M	0.401	1.248	20.14	ns
		Repeated vs. No TIV	D0	0.848	0.733	55.93	ns
			12M	0.106	1.962	58.94	ns
		First TIV 2013 vs. No TIV	D0	0.865	0.482	21.96	ns
			12M	**0.030**	2.791	24.08	#
		Pre-2010 Only vs. No TIV	D0	0.924	0.096	39.08	ns
			12M	0.864	0.172	43.60	ns
	A/H3N2						
		Repeated (n = 33)	D0 vs. D21	**<0.001**	8.744	30.00	***
			D0 vs. 3M	**<0.001**	8.343	31.00	***
			D0 vs. 6M	**<0.001**	5.702	31.00	***
			D0 vs. 12M	0.233	1.216	31.00	ns
		First TIV 2013 (n = 11)	D0 vs. D21	**0.006**	4.376	10.00	**
			D0 vs. 3M	**0.007**	4.008	10.00	**
			D0 vs. 6M	**0.014**	3.372	10.00	*
			D0 vs. 12M	0.055	2.171	10.00	ns
		Pre-2010 Only (n = 27)	D0 vs. 12M	0.434	0.7977	20.00	ns
		No TIV (n = 39)	D0 vs. 12M	0.060	1.940	28.00	ns
		Repeated vs. First TIV 2012	D0	**0.002**	4.754	12.73	##
			D21	0.954	0.2789	9.472	ns
			3M	0.954	0.2777	10.50	ns
			6M	0.803	0.8423	10.40	ns

Analysis				Adjusted p value	t value	Degree of freedom	Summary
			12M	0.389	1.700	11.56	ns
		Repeated vs. No TIV	D0	**<0.001**	7.279	55.93	###
			12M	**<0.001**	6.062	58.94	###
		First TIV 2013 vs. No TIV	D0	0.193	1.121	14.50	ns
			12M	**0.017**	2.936	16.04	#
		Pre-2010 Only vs. No TIV	D0	**0.037**	2.474	32.94	#
			12M	**0.017**	2.755	40.61	#
	B						
		Repeated (n = 33)	D0 vs. D21	**<0.001**	5.943	30.00	***
			D0 vs. 3M	**0.004**	3.578	31.00	**
			D0 vs. 6M	0.449	1.152	31.00	ns
			D0 vs. 12M	0.449	1.104	31.00	ns
		First TIV 2013 (n = 11)	D0 vs. D21	**<0.001**	6.119	10.00	***
			D0 vs. 3M	**0.001**	4.627	10.00	***
			D0 vs. 6M	**0.006**	3.575	10.00	**
			D0 vs. 12M	**0.032**	2.359	10.00	*
		Pre-2010 Only (n = 27)	D0 vs. 12M	0.363	0.9309	20.00	ns
		No TIV (n = 39)	D0 vs. 12M	**0.044**	2.106	29.00	*
		Repeated vs. First TIV 2012	D0	0.890	0.4373	15.95	ns
			D21	**0.048**	2.116	18.71	#
			3M	**0.048**	2.111	19.47	#
			6M	0.103	1.439	17.29	ns
			12M	0.168	1.234	16.84	ns
		Repeated vs. No TIV	D0	**<0.001**	4.133	67.97	###
			12M	**<0.001**	3.986	61.86	###
		First TIV 2013 vs. No TIV	D0	**0.006**	3.190	16.50	##
			12M	**<0.001**	4.026	17.54	###
		Pre-2010 Only vs. No TIV	D0	**0.002**	3.548	44.69	##
			12M	**<0.001**	3.826	46.94	###

Data was log-transformed and analysed using mixed-effects model with the Geisser-Greenhouse correction and Holm-Sidak's multiple comparisons test with individual variances computed for each comparison.
ns – not significant p value > 0.05

Supplementary Table 13: Statistical analysis for Figure 6 and Supplementary Figure 2.

Analysis	Adjusted p value	t value[1]	Degree of freedom[1]	z score[2]	Summary
**Fig. 6A – HI titres D0**					
A/H1N1pdm09 (CA09)					
1st (pdm) vs. 2nd	**<0.001**	12.29	132.0	-	***
1st (pdm) vs. 3rd	**<0.001**	10.74	78.00	-	***
1st (pdm) vs. 4th	**<0.001**	9.376	55.00	-	***
1st (pdm) vs. 5th	**<0.001**	5.570	32.00	-	***
2nd vs. 3rd	**<0.001**	4.722	78.00	-	***
2nd vs. 4th	**<0.001**	5.216	55.00	-	***
2nd vs. 5th	0.152	1.813	32.00	-	ns
3rd vs. 4th	0.098	2.179	54.00	-	ns
3rd vs. 5th	0.947	0.067	32.00	-	ns
4th vs. 5th	**0.037**	2.761	32.00	-	*
A/H3N2					
1st vs. 2nd PE09	**<0.001**	3.920	48.00	-	***
1st PE09 vs. 1st VI11 (+PE09)	**<0.001**	8.354	68.00	-	***
1st PE09 vs. 2nd VI11 (+PE09)	**<0.001**	7.354	45.00	-	***
2nd PE09 vs. 1st VI11 (+PE09)	**<0.001**	4.387	41.00	-	***
2nd PE09 vs. 2nd VI11 (+PE09)	**0.005**	3.291	32.00	-	**
1st vs. 2nd VI11 (+PE09)	0.087	1.750	43.00	-	ns
1st VI11 (+PE09) vs. 1st VI11 only	**0.002**	-	-	3.240	**
2nd VI11 (+PE09) vs. 1st VI11 only	**<0.001**	-	-	3.579	**
B					
1st vs. 2nd B/Vic	**0.044**	2.365	48.00	-	*
1st B/Vic vs. 1st B/Yam (+B/Vic)	0.879	0.153	69.00	-	ns
1st B/Vic vs. 2nd B/Yam (+B/Vic)	**0.002**	3.655	45.00	-	**
2nd B/Vic vs. 1st B/Yam (+B/Vic)	0.085	2.296	42.00	-	ns
2nd B/Vic vs. 2nd B/Yam (+B/Vic)	0.265	1.502	32.00	-	ns
1st vs. 2nd B/Yam (+B/Vic)	**<0.001**	6.694	45.00	-	***
1st B/Yam (+B/Vic) vs. 1st B/Yam only	>0.999	-	-	0.317	ns
2nd B/Yam (+B/Vic) vs. 1st B/Yam only	0.143	-	-	1.802	ns
**Fig. 6B – HI titres D21**					
A/H1N1pdm09 (CA09)					
1st (pdm) vs. 2nd	**<0.001**	5.071	129.0	-	***
1st (pdm) vs. 3rd	**<0.001**	5.813	76.00	-	***
1st (pdm) vs. 4th	**<0.001**	7.139	55.00	-	***
1st (pdm) vs. 5th	**<0.001**	5.813	31.00	-	***
2nd vs. 3rd	0.157	1.429	76.00	-	ns
2nd vs. 4th	**<0.001**	4.227	55.00	-	***
2nd vs. 5th	**<0.001**	5.191	31.00	-	***
3rd vs. 4th	**<0.001**	5.191	52.00	-	***
3rd vs. 5th	**<0.001**	6.581	29.00	-	***
4th vs. 5th	**<0.001**	4.746	31.00	-	***
A/H3N2					
1st vs. 2nd PE09	0.979	0.078	45.00	-	ns
1st PE09 vs. 1st VI11 (+PE09)	**<0.001**	4.378	69.00	-	***
1st PE09 vs. 2nd VI11 (+PE09)	**0.021**	2.827	44.00	-	*

Analysis			Adjusted p value	t value[1]	Degree of freedom[1]	z score[2]	Summary
		2nd PE09 vs. 1st VI11 (+PE09)	**<0.001**	5.476	40.00	-	***
		2nd PE09 vs. 2nd VI11 (+PE09)	**0.002**	3.947	29.00	-	**
		1st vs. 2nd VI11 (+PE09)	0.979	0.185	43.00	-	ns
	B						
		1st vs. 2nd B/Vic	**0.005**	3.595	47.00	-	**
		1st B/Vic vs. 1st B/Yam (+B/Vic)	0.722	0.728	70.00	-	ns
		1st B/Vic vs. 2nd B/Yam (+B/Vic)	0.502	1.429	44.00	-	ns
		2nd B/Vic vs. 1st B/Yam (+B/Vic)	0.722	0.950	41.00	-	ns
		2nd B/Vic vs. 2nd B/Yam (+B/Vic)	0.383	1.741	30.00	-	ns
		1st vs. 2nd B/Yam (+B/Vic)	0.722	0.806	44.00	-	ns
**Fig. 6C – Half-life from D21 (months)**							
	A/H1N1pdm09 (CA09)						
		1st (pdm) vs. 2nd	**<0.001**	10.50	116.0	-	***
		1st (pdm) vs. 3rd	**<0.001**	10.25	74.00	-	***
		1st (pdm) vs. 4th	**<0.001**	11.09	52.00	-	***
		1st (pdm) vs. 5th	**<0.001**	6.490	27.00	-	***
		2nd vs. 3rd	**0.042**	2.410	68.00	-	*
		2nd vs. 4th	**0.001**	4.055	50.00	-	**
		2nd vs. 5th	**0.019**	3.166	26.00	-	*
		3rd vs. 4th	0.362	1.503	49.00	-	ns
		3rd vs. 5th	0.362	1.396	25.00	-	ns
		4th vs. 5th	0.570	0.575	26.00	-	ns
	A/H3N2						
		1st vs. 2nd PE09	0.365	1.652	35.00	-	ns
		1st PE09 vs. 1st VI11 (+PE09)	0.365	1.596	56.00	-	ns
		1st PE09 vs. 2nd VI11 (+PE09)	**0.011**	3.374	36.00	-	*
		2nd PE09 vs. 1st VI11 (+PE09)	0.515	0.659	33.00	-	ns
		2nd PE09 vs. 2nd VI11 (+PE09)	0.482	1.103	25.00	-	ns
		1st vs. 2nd VI11 (+PE09)	0.154	2.219	36.00	-	ns
		1st VI11 (+PE09) vs. 1st VI11 only	0.304	-	-	1.433	ns
		2nd VI11 (+PE09) vs. 1st VI11 only	**0.033**	-	-	2.394	*
	B						
		1st vs. 2nd B/Vic	0.761	0.646	41.00	-	ns
		1st B/Vic vs. 1st B/Yam (+B/Vic)	0.761	1.124	59.00	-	ns
		1st B/Vic vs. 2nd B/Yam (+B/Vic)	0.761	0.976	40.00	-	ns
		2nd B/Vic vs. 1st B/Yam (+B/Vic)	0.556	1.567	33.00	-	ns
		2nd B/Vic vs. 2nd B/Yam (+B/Vic)	0.761	1.178	27.00	-	ns
		1st vs. 2nd B/Yam (+B/Vic)	0.761	0.903	36.00	-	ns
**S.Fig. 5 – Foldchange D21/D0**							
	A/H1N1pdm09 (CA09)						
		1st (pdm) vs. 2nd	**<0.001**	13.14	128.0	-	***
		1st (pdm) vs. 3rd	**<0.001**	12.01	72.00	-	***
		1st (pdm) vs. 4th	**<0.001**	13.04	54.00	-	***
		1st (pdm) vs. 5th	**<0.001**	10.25	31.00	-	***
		2nd vs. 3rd	**<0.001**	4.591	71.00	-	***
		2nd vs. 4th	**<0.001**	6.898	54.00	-	***
		2nd vs. 5th	**<0.001**	5.248	31.00	-	***
		3rd vs. 4th	**<0.001**	4.489	48.00	-	***

Analysis	Adjusted p value	t value[1]	Degree of freedom[1]	z score[2]	Summary
3rd vs. 5th	**<0.001**	4.696	29.00	-	***
4th vs. 5th	0.6941	0.397	30.00	-	ns
A/H3N2					
1st vs. 2nd PE09	**0.042**	2.094	44.00	-	*
1st PE09 vs. 1st VI11 (+PE09)	**0.006**	3.413	65.00	-	**
1st PE09 vs. 2nd VI11 (+PE09)	**<0.001**	5.352	44.00	-	***
2nd PE09 vs. 1st VI11 (+PE09)	0.861	0.489	39.00	-	ns
2nd PE09 vs. 2nd VI11 (+PE09)	0.901	0.125	29.00	-	ns
1st vs. 2nd VI11 (+PE09)	0.118	2.234	41.00	-	ns
1st VI11 (+PE09) vs. 1st VI11 only	**0.034**	-	-	2.635	*
2nd VI11 (+PE09) vs. 1st VI11 only	**<0.001**	-	-	3.776	***
B					
1st vs. 2nd B/Vic	**0.043**	2.259	44.00	-	*
1st B/Vic vs. 1st B/Yam (+B/Vic)	0.922	0.360	63.00	-	ns
1st B/Vic vs. 2nd B/Yam (+B/Vic)	0.111	1.977	44.00	-	ns
2nd B/Vic vs. 1st B/Yam (+B/Vic)	**0.007**	3.445	39.00	-	**
2nd B/Vic vs. 2nd B/Yam (+B/Vic)	0.922	0.236	29.00	-	ns
1st vs. 2nd B/Yam (+B/Vic)	**0.001**	4.203	39.00	-	***
1st B/Yam (+B/Vic) vs. 1st B/Yam only	>0.999	-	-	0.332	ns
2nd B/Yam (+B/Vic) vs. 1st B/Yam only	**0.037**	-	-	2.502	*

[1]Data was log-transformed and analysed using mixed-effects model with the Geisser-Greenhouse correction and Holm-Sidak's multiple comparisons test with individual variances computed for each comparison. Adjusted p value, t value, and degree of freedom are reported.

[2]Data between participants who were vaccinated with A/H3N2 VI11 (+PE09) or B/Yam (+B/Vic) and those who were vaccinated with A/H3N2 VI11 only or B/Yam only were analysed using nonparametric Kruskal-Wallis test and Dunn's multiple comparisons test. Adjusted p values and z score are reported.

ns – not significant p value > 0.05
